# A robust ambient temperature collection and stabilization strategy: Enabling worldwide functional studies of the human microbiome

**DOI:** 10.1038/srep31731

**Published:** 2016-08-25

**Authors:** Ericka L. Anderson, Weizhong Li, Niels Klitgord, Sarah K. Highlander, Mark Dayrit, Victor Seguritan, Shibu Yooseph, William Biggs, J. Craig Venter, Karen E. Nelson, Marcus B. Jones

**Affiliations:** 1 Human Longevity, Inc., San Diego, CA 92121, USA; 2 Genomic Medicine, J. Craig Venter Institute, La Jolla, CA 92037, USA.

## Abstract

As reports on possible associations between microbes and the host increase in number, more meaningful interpretations of this information require an ability to compare data sets across studies. This is dependent upon standardization of workflows to ensure comparability both within and between studies. Here we propose the standard use of an alternate collection and stabilization method that would facilitate such comparisons. The DNA Genotek OMNIgene∙Gut Stool Microbiome Kit was compared to the currently accepted community standard of freezing to store human stool samples prior to whole genome sequencing (WGS) for microbiome studies. This stabilization and collection device allows for ambient temperature storage, automation, and ease of shipping/transfer of samples. The device permitted the same data reproducibility as with frozen samples, and yielded higher recovery of nucleic acids. Collection and stabilization of stool microbiome samples with the DNA Genotek collection device, combined with our extraction and WGS, provides a robust, reproducible workflow that enables standardized global collection, storage, and analysis of stool for microbiome studies.

Advances in DNA sequencing have enabled researchers to broadly assess microbial communities in a culture-independent, high-resolution manner. Metagenomic studies have been used to monitor shifts in human microbiome composition and function associated with diseases such as obesity^1^, diabetes^2^, and cancer^3^. Recent discoveries have suggested the utility of microbiome profiles as biomarkers with diagnostic and prognostic value^4^,^5^. It is evident, however, that the validity and reproducibility of data from these studies are highly dependent upon the quality of the collected microbiome samples. For any analysis, an accurate microbiome snapshot must be captured when the sample is collected. Key to this is immediate stabilization of the sample. Although one study claimed no differences were detected after two weeks storage at room temperature^6^, many studies^7^,^8^,^9^,^10^,^11^,^12^ have demonstrated the importance of proper storage of these samples for accurate results.

Recently, we reported on how sequencing library preparation artifacts can influence microbiome data^13^, highlighting the need for consistent and standardized protocols to improve the interpretation of microbiome data. There have also been recent calls from the larger scientific community for a standardized global study to assess the diversity of the human microbiome and correlations with health and disease^14^. In order for such a study to be feasible, thousands of microbiome samples would need to be collected, stabilized in remote locations, and shipped to the laboratory for processing. Ambient temperature storage and shipment of samples would greatly facilitate and standardize such a study by enabling easy collection outside of the clinic, avoiding inconsistent sample handling and storage, and reducing shipping costs.

A recent study^15^ used 16S rDNA sequencing to compare a new commercially available ambient temperature stabilization kit, OMNIgene∙GUT (DNA Genotek, Inc. Ottawa, CAN), to stabilization in RNAlater (Ambion, Austin, TX), in Tris-EDTA buffer, and storage by freezing using repeated sampling from a single subject. The authors proposed use of OMNIgene∙GUT as an alternative stabilization method when refrigeration and cold-chain transportation are not available. We chose to extend this analysis to a larger subject population and to use whole genome sequencing (WGS) to analyze the microbial communities in each sample, as the impact of storage conditions can vary. WGS increases the resolution and specificity of metagenomic analyses compared to 16S rDNA sequencing^16^,^17^, and enables gene and functional analysis. We elected to compare freshly extracted samples without storage (Basal) to DNA Genotek stabilized samples (Stabilized) and to samples that were frozen at −20 °C (Frozen), emphasizing conditions that are more readily available globally.

## Results

### Nucleic acid output per mg stool is consistently higher in Stabilized samples

**Figure 1:**
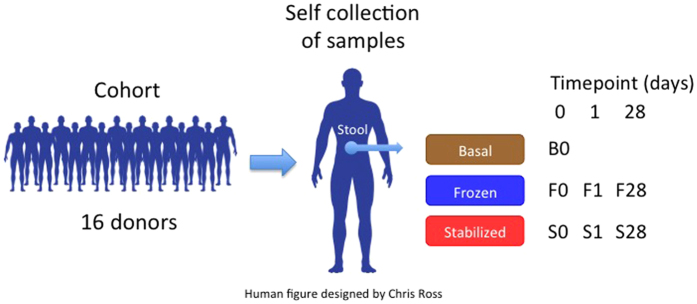
Experimental Design. A cohort of 16 volunteer donors was recruited for self collection of stool microbiome samples. Stool samples were aliquoted into Basal, Frozen, and Stabilized aliquots with time points taken as marked.

**Figure 2:**
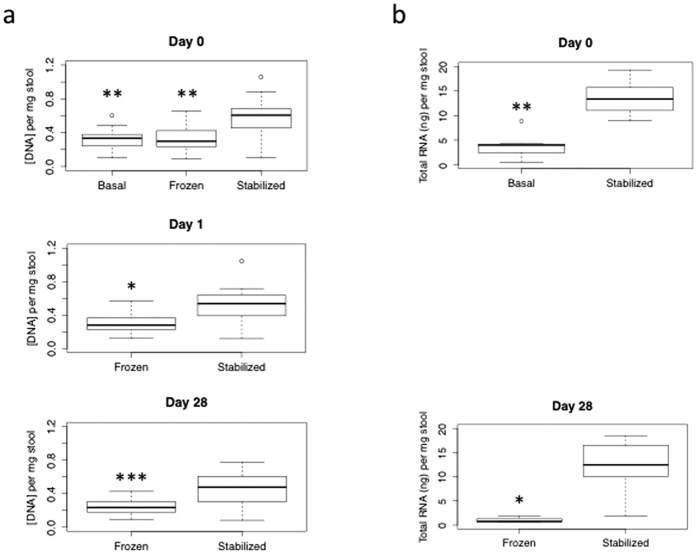
Stabilized samples have increased nucleic acid yields. Box whisker plots of yields of (**a**) DNA (ng/μl) per mg stool input on Day 0, Day 1, and Day 28. (**b**) Total RNA (ng)/mg stool input at Day 0 and Day 28. Day 28 RNA Frozen (N = 5). Wilcoxon Rank Sum Test: *p-value < 0.01, **p-value < 0.001, ***p-value < 0.0001.

Donor stool samples were collected and DNA extractions were performed as described (see Methods, Fig. 1). Throughout the time course, the amount of DNA recovered per mg stool input was significantly higher in Stabilized samples compared to both Basal (freshly extracted without storage) and Frozen samples. Stabilized samples on Day 0 recovered on average 79% more DNA than Basal samples and 74% more DNA than Frozen samples (*p-*values 0.0009 and 0.001 respectively). DNA recovered from Stabilized samples on Day 1, and Day 28 were also significantly higher than Frozen samples, with *p*-values of 0.004 and 0.0001 respectively, and on average 65% and 79% more DNA recovered (Fig. 2a). Consistent with DNA extractions, RNA extracted from Stabilized samples also gave higher total RNA recovered per mg stool input. On average, Stabilized samples yielded 250% more RNA than Basal samples on Day 0, and 1000% more RNA after 28 days freezer storage (Fig. 2b). These extractions were significantly different, with *p*-values of 0.0006 and 0.007 respectively. High quality cDNA libraries were made reproducibly using RNA extracted from Stabilized samples, indicating that the RNA extracted was of high quality. Sequencing metrics from RNASeq libraries extracted from a Stabilized stool sample in duplicate are shown in Supplemental Table S1. These results indicate Stabilized samples yield significantly higher DNA and RNA than Frozen samples.

### Assembly and sequencing metrics are largely unaffected by stabilization method

Sequencing metrics were compared to examine for any biases associated with the stabilization methods (Table 1, Supplementary Table S2). The summary of sequencing assembly metrics is shown in Supplementary Table S3. In order to ensure an unbiased comparison, we used the same number of non-human reads for assembly of all samples. In all sequencing assembly metrics tested, except for % human reads, no significant differences were detected (Supplementary Table S2, *p*-values > 0.05: see Supplemental Table S4). However the % human reads in Stabilized samples were significantly lower than Frozen samples, albeit a small decrease (0.04% vs 0.06%, *p*-value 0.00576). For each individual sample, the assembly quality was assessed by considering total contig length *vs*. contig rank in the order of decreasing length (Supplementary Fig. S1). We did not detect any patterns based on stabilization method. Within sequencing metrics, the % duplicated reads was significantly lower in Stabilized samples, compared to both Basal and Frozen samples (*p*-values 0.02964 and 0.00139 respectively), and the fraction of low quality reads was lower in Stabilized samples compared to Frozen samples (*p*-value 0.03473). These results indicate that the sequencing and assembly results for Stabilized and Frozen methods largely are comparable, with the exceptions of the observed decreases in % human reads and % duplicated reads.

### Stabilized samples yield taxonomic and abundance profiles consistent with Basal and Frozen signatures

**Figure 3:**
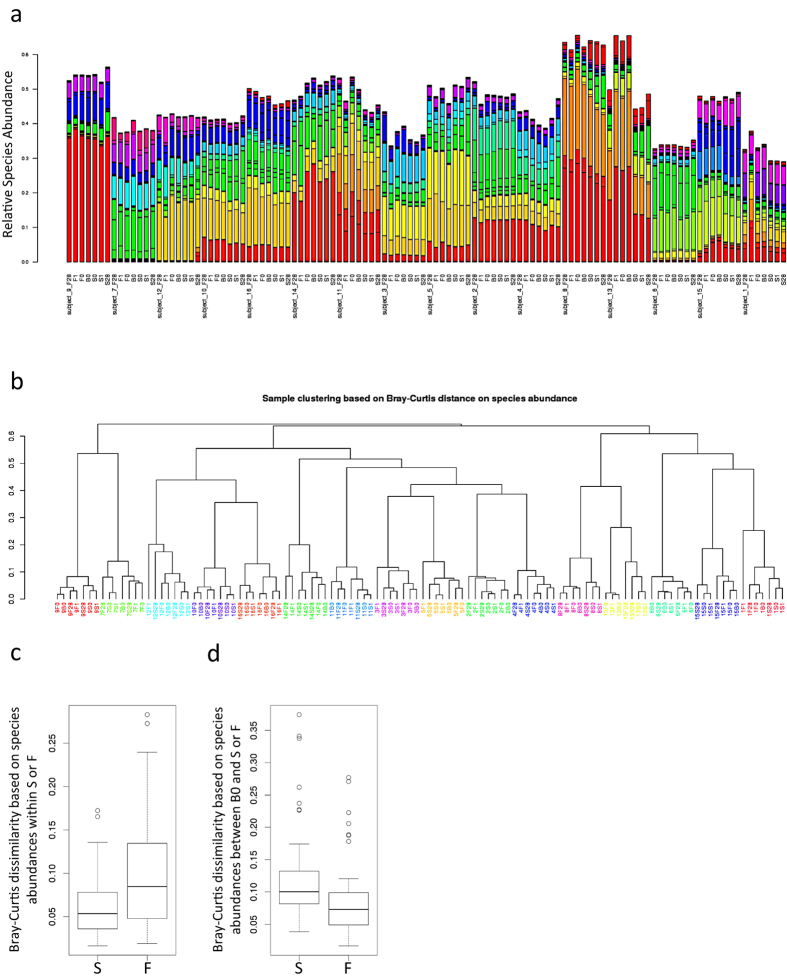
Stabilized samples are consistent with Frozen and Basal samples. (**a**) Relative species abundances of the top 30 species across all 16 subjects were plotted for each individual sample from each subject. (**b**) Bray-Curtis dissimilarity based on relative species abundances was used to cluster all samples. Each color represents a distinct subject. (**c**) Box and whisker plots of calculated pairwise Bray-Curtis dissimilarity within either Frozen or Stabilized. (**d**) Box and whisker plots of calculated pairwise Bray-Curtis dissimilarity between Basal and either Frozen or Stabilized. B0: Basal; F0, F1, F28: Frozen Day 0, Day 1, and Day 28; S0, S1, S28: Stabilized Day 0, Day 1, and Day 28; F: Frozen; S: Stabilized.

An important aspect of microbiome collection and storage protocols is reproducibility. Relative species abundances of the top thirty species across all subjects were compared in each sample to determine the reproducibility of Stabilized samples to Basal and Frozen samples (Fig. 3a). In the majority of subjects, both Frozen and Stabilized samples retained similar species abundances to those of the corresponding Basal sample, with the exception of subject 13 for Stabilized samples, and individual timepoints for Frozen samples in subjects 1 and 13. Individual species abundance graphs for all subjects are shown in Supplemental Fig. S2. Relative species abundances from each time point of the Frozen and Stabilized samples were compared to the relative species abundances of the Basal sample using the Bray-Curtis dissimilarity scores. Clustering based on these dissimilarity scores revealed that intra-subject variation was greater than changes based on stabilization method, as each subject clustered into its own clade, and these subject clades had short branch lengths (Fig. 3b). Bray-Curtis dissimilarity scores within Frozen samples ranged from 0.019 to 0.283, while dissimilarity scores within Stabilized samples had a narrower range from 0.016 to 0.172 (Fig. 3c). Both Frozen and Stabilized samples remained similar to the Basal sample with low Bray-Curtis dissimilarity score medians of 0.073 and 0.1 respectively (Fig. 3d), indicating results with Stabilized samples are consistent with those of Frozen samples.

In healthy adults, 80% of fecal taxa fall within three phyla: Bacteroidetes, Firmicutes, and Actinobacteria^18^. The fold change in Frozen and Stabilized Bacteroidetes and Firmicutes phylum abundance relative to Basal was quantified in order to compare the degree of change in Stabilized samples. The Bacteroidetes phylum remained stable in both Frozen and Stabilized samples, with an average fold change of 0-fold across all time points relative to Basal (Supplemental Fig. S3a,b, Supplemental Table S5). The Firmicutes phylum remained stable in Frozen samples with an average of 0-fold across all samples, while the average was 0.2-fold in Stabilized samples (Supplemental Fig. S3a,b, Supplemental Table S5). Comparing Frozen fold-changes to Stabilized fold-changes, the Day 1 Firmicutes and Day 28 fold changes for both Firmicutes and Bacteroidetes were significantly different (*p*-values: 0.03864, 0.0155, and 0.01825 respectively), but this was not the case for Day 0 (*p*-values: 0.6772 and 0.06396 for Day 0 Firmicutes and Bacteroidetes respectively). However these incremental changes are unlikely to have a biological effect.

**Figure 4:**
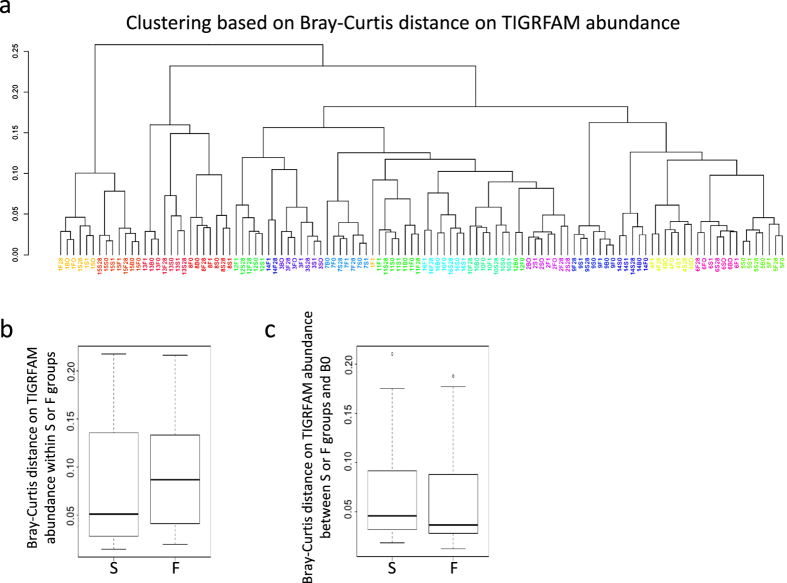
Stabilized samples maintain results of TIGRFAM analysis. (**a**) Bray-Curtis distances based on relative TIGRFAM abundances were used to cluster all samples. Each color represents a distinct subject. (**b**) Box and whisker plots of calculated pairwise Bray-Curtis dissimilarity within either Frozen or Stabilized. (**c**) Box and whisker plots of calculated pairwise Bray-Curtis dissimilarity between Basal and either Frozen or Stabilized. B0: Basal; F0, F1, F28: Frozen Day 0, Day 1, and Day 28; S0, S1, S28: Stabilized Day 0, Day 1, and Day 28; F: Frozen; S: Stabilized.

Individual species within a microbiome can perform the same function(s) as another individual species, which would be masked in a purely taxonomic analysis. Therefore characterizing the functional capability of a microbiome at the protein level is critical. Taking advantage of WGS, we compared relative TIGRFAM^19^ and Pfam^20^ abundances from each time point of Basal, Frozen, and Stabilized samples to determine the similarity of stabilized samples to Basal using the Bray-Curtis dissimilarity scores. Consistent with the taxonomic based distances, clustering based on these Bray-Curtis dissimilarity scores revealed intra-subject variation was greater than changes based on stabilization method, as the majority of subjects clustered into subject clades, and these subject clades had short branch lengths (Fig. 4a, Supplemental Fig. S4a). However, in subjects 1 and 12 for TIGRFAM, and subjects 1, 12, and 14 for Pfam, the time points/conditions were split across 2 distinct clades. TIGRFAM Bray-Curtis dissimilarity within Frozen samples ranged from 0.019 to 0.216 while distances within Stabilized samples ranged from 0.014 to 0.218 (Fig. 4b) indicating low variability within both stabilization methods. Both Frozen and Stabilized samples remained similar to Basal with low Bray-Curtis distance score medians of 0.038 and 0.046 respectively (Fig. 4c), indicating Stabilized samples give consistent results to that of Frozen samples.

### Stabilized and Frozen samples are reproducible

**Figure 5:**
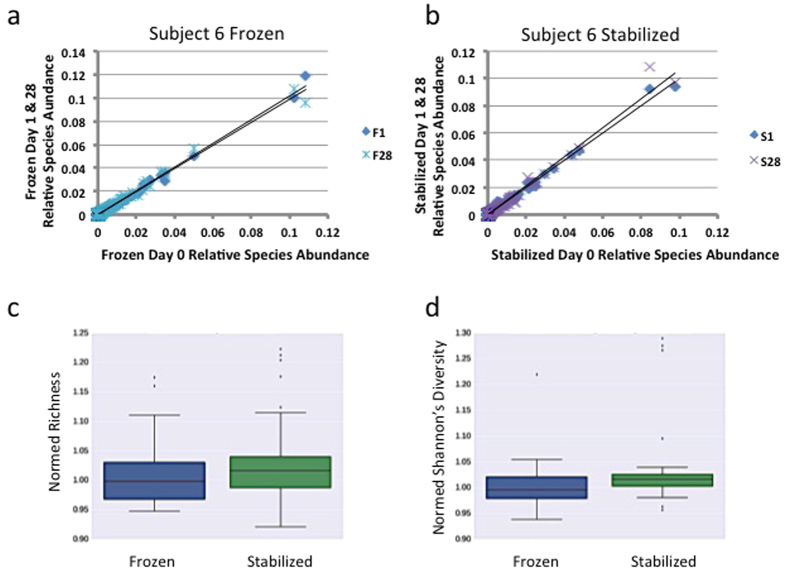
Stabilized and Frozen samples are reproducible. Precision analysis of (**a**) Frozen Day 0 relative species abundances plotted against corresponding Frozen Day 1 and Day 28 relative species abundances in representative subject 6, (**b**) Stabilized Day 0 relative species abundances plotted against corresponding Stabilized Day 1 and Day 28 relative species abundances in representative subject 6. Black line = linear regression analysis. R^2^ values for all subjects are in Table 2. (c) Boxplot of normalized Frozen and Stabilized richness scores across all subjects and time points. (**d**) Boxplot of normalized Frozen and Stabilized diversity (SDI) scores across all subjects and time points.

Ideally, metagenomic analyses of properly stabilized samples should produce identical data, regardless of the length of time that the sample is stored. In order to address the reproducibility of Freezing and Stabilized methods with time in storage, precision analysis was used to compare within a given method the relative species abundance values for Day 0, to the Day 1 and to the Day 28 relative species abundance values. Linear regression analysis was used to determine how well the line explains the data, with r^2^ = 1 indicating a perfect fit. In representative Subject 6, within Frozen samples, relative species abundance values of Day 0 compared to both Day 1 and Day 28 retain high reproducibility, with r^2^ values of 0.99 (Fig. 5a, Table 2). Precision analysis in representative Subject 6 also showed high reproducibility in Stabilized samples, with r^2^ values of 0.98 and 0.99 (Fig. 5b, Table 2). Regression values for all subjects comparing Day 0 to both Day 1 and Day 28 relative species abundances are listed in Table 2. These results indicate Stabilized sample reproducibility is consistent with reproducibility found in Frozen samples.

Species richness and biodiversity are vital to the function of a microbiome and decreases in both are correlated with disease in humans^21^. Richness was calculated by counting the number of distinct species found in the sample above a set threshold of relative species abundance and read count. To assess biodiversity we used Shannon’s Diversity index (SDI), which takes into account both the number of distinct species, and the evenness of those species by accounting for the relative species abundances. Comparing samples within a method, both Frozen and Stabilized samples had similar richness and SDI scores across time points (Supplemental Table S6). Using aggregate data that includes all subjects, no significant difference was observed between Frozen and Stabilized community richness (Fig. 5c, p-values > 0.1 see Supplementary Table S6), however Stabilized samples had significantly, albeit very small, increased SDI scores (Fig. 5d, *p*-value 0.00171, Supplemental Table S6). These results indicate Stabilized samples richness are consistent with those of Frozen samples and biodiversity may be increased in Stabilized samples.

## Discussion

Stabilization of biological samples is crucial for accurate analysis of microbiome data, and for comparison across studies. Unstabilized samples introduce unwanted variation due to changes in the community caused by differential cell growth or death and the potential for nucleic acid degradation that can result in taxonomic predictions that do not accurately reflect the *in vivo* community at the time of sample collection. Current sample collection protocols from the NIH funded Human Microbiome Project (HMP) recommend sample storage at −80 °C^22^, while the Center for Disease Control recommends storage below −15 °C^23^. These recommendations limit the ability to perform sample collections at geographic locations where access to these storage conditions do not exist, and limit collections from large cohorts that require storage of a large number of samples at these temperatures. In order for samples to be shared across laboratories, specimens undergo multiple freeze/thaw cycles or require an additional burden of aliquoting and archiving samples. In order to collect a large global reference set as proposed^14^, it will be important to implement a standard collection protocol that is not restricted by location, by availability of required storage conditions, nor by limitations on sample sharing.

In this study, the ability of the DNA Genotek OMNIgene∙Gut Stool Microbiome Kit (Stabilized) to stabilize and store stool samples was compared to fresh stool samples (Basal) as a reference point, and the standard storage protocol of freezing (Frozen). Stabilized samples were found to be equivalent or better than Frozen samples in all technical metrics tested, yet added additional functionality. Stabilized samples yielded higher quantities of both DNA and RNA, thereby decreasing the amount of stool needed for further analysis. This is especially important for mRNA sequencing, since >90% of the RNA is ribosomal and is discarded during processing. Stabilized and Frozen samples provided both unbiased and reproducible results comparable to those of the Basal sample based on measures of sequencing metrics, species abundance, functional profiles, and diversity measures. We also did not detect significant change between Stabilized and Frozen samples. Intra-subject variation was greater than changes based on stabilization method. These findings are consistent with recent work comparing this kit to other stabilization methods^15^,^24^.

The majority of human microbiome characterization has focused on microbial community structure, the types and numbers of microbes present, but recently there is a transition towards a focus on microbial community function–the metabolic activities and end products resulting from microbial activity^25^. The application of WGS to microbiome characterization enables this functional level analysis, increasing the usefulness of microbiome analysis to the clinic. Compared to 16S rDNA sequencing at the same sequencing depth, WGS also increases resolution and specificity^16^,^17^, resulting in increased coverage of low abundant organisms, and enabling of gene level analysis. Progress towards medically actionable outcomes for the microbiome will depend on the characterization of the microbiome at the functional level, requiring WGS in future standardized workflows.

Global implementation of the DNA Genotek kit, or a similar collection/stabilization device, would present the microbiome community with increased consistency and standardization of sample collection and provides an automation-friendly device that enables high-throughput processing. This kit removes the need for expensive dry ice and cold pack shipping, as well as reduces the carbon footprint of −20 °C/−80 °C freezers, by permitting storage at ambient temperature. This device enables transfer of identical sample material between community members, as samples are immediately stabilized and homogenized, and aliquots can be taken at ambient temperature, removing the hindrance of freeze/thaw cycles. These properties enable collaborative studies, including laboratories in distinct geographical sites across the world, reducing the potential for location specific sample collection effects, and reducing costs. Together, the implementation of DNA Genotek stabilization with WGS enables functional characterization of global microbiomes.

## Methods

### Experimental Design

Stool samples were collected from consented donors under an IRB approved protocol. Upon collection, samples were aliquoted into three major treatments: Basal: freshly extracted without stabilization or storage, Frozen: storage at −20 °C, and Stabilized: stabilized by storage in the OMNIgene∙Gut stabilization kit (DNA Genotek, Ontario, CAN) (Fig. 1). Basal samples were prepared for DNA extraction upon collection. Frozen samples were aliquoted into 1 tube per time point on Day 0 and then immediately frozen at −20 °C. At each time point, an individual sample tube was thawed and then prepared for nucleic acid extraction. Stabilized samples were transferred into a DNA Genotek stabilization tube and immediately mixed with stabilization reagent. An aliquot was taken at each time point for extraction. The research objectives of this study were to compare the stabilization effects of the DNA Genotek stabilization reagent to freezing, using freshly extracted material (Basal) as a reference point. Metagenomic sequencing was undertaken using DNA extracted from each de-identified stool sample.

### Ethics, consent, and permissions

This study was conducted under a protocol approved by the Western Institutional Review Board (HLI 2014–002), informed consent was obtained from all subjects, and work was done in accordance with the approved protocol. Based on the restrictions set by the IRB, raw sequences are stored in the HLI Knowledgebase. Donated fecal samples were collected from 16 donors.

### DNA extraction

#### Basal and Frozen Stool Samples

Lysis buffer (5 mL, Perkin Elmer 852) was added to each stool sample (0.5 g) and vortexed until homogenous. Homogenized sample (1.2 mL) and Proteinase K (15 μL, Sigma Aldrich, PN. P2308) enzyme was aliquoted to a 1.5 mL tube with garnet beads (Mo Bio PN. 12830-50-BT).

#### Stabilized Stool Samples

Stool sample (0.5 g) was added to the stabilization tube and the tube was then shaken vigorously, following manufacturer’s protocol. Lysis buffer (900 μL, 20 mM Tris-HCl pH 8.0, 2 mM Sodium EDTA 1.2% Triton X-100), Stabilized sample (300 μL), and Proteinase K (15 μL, Sigma Aldrich, PN. P2308) enzyme was aliquoted to a 1.5 mL tube with garnet beads (Mo Bio PN. 12830-50-BT).

#### All samples

Bead tubes were then incubated at 65 °C for 10 minutes and then 95 °C for 10 minutes. Tubes were then placed in a Vortex Genie 2 to perform bead beating for 13 minutes and the sample subsequently spun in an Eppendorf Centrifuge 5424. Supernatant (700 μL) was then transferred to a deep well block. DNA extraction and purification was performed using a Chemagic MSM I (Perkin Elmer) following the manufacturer’s protocol. Samples were then further purified using the Onestep Inhibitor Removal kit following manufacturer’s instructions (Zymo Research PN. D6035). DNA samples were then quantified using Quant-iT on an Eppendorf AF2200 plate reader.

### RNA extraction

The same extraction protocol was performed as with DNA extraction, with the following changes: the Perkin Elmer 1076 lysis buffer was used. After extraction on Chemagic MSM I, removal of DNA and concentrating of RNA was performed using Zymo RNA Clean and Concentrator kit (Zymogen Research R1014). RNA was quantified using Quant-iT on an Eppendorf AF2200 plate reader, measuring nucleic acid concentration and optical density at 260 nm and 280 nm.

### Nextera XT Library Preparation

Libraries were prepared manually following the manufacturer’s protocol (Illumina, PN. 15031942). Briefly, samples were normalized to 0.2 ng/μl DNA material per library using a Quant-iT picogreen assay system (Life Technologies, PN. Q33120) on an AF2200 plate reader (Eppendorf), then fragmented and tagged via tagmentation. Amplification was performed by Veriti 96 well PCR (Applied Biosystems) followed by AMPure XP bead cleanup (Beckman Coulter, PN. A63880). Fragment size was measured using Labchip GX Touch high-sensitivity.

### Cluster Generation and Next Generation Sequencing

Samples were normalized to 1 nM, pooled, and diluted to 8 pM. The paired-end cluster kit V4 was used and cluster generation was performed on an Illumina cBot, with pooled samples in all 8 lanes. Sequencing was performed on an Illumina HiSeq 2500 using SBS kit V4 chemistry. Median Cluster densities (K mm^2^) were 908.5 for Nextera XT.

### Taxonomic assignment and assembly analysis

Microbiome sequences were processed and analyzed with Human Longevity Inc’s (HLIs) proprietary microbiome annotation pipeline. Raw BCL data were de-multiplexed and converted to Paired End (PE) reads of 2 × 125 base pairs in FASTQ format, trimming the adapter sequence. Reads were then filtered using Trimmomatic^26^. After removal of low quality bases, and reads shorter than 90 nt, duplicated read pairs were identified with the program cd-hit-dup^27^ by matching the first 50 bases from both R1 and R2 reads (cd-hit-dup parameter -u 50).

Reads were aligned to Human genome hg38 using BWA^28^ and all reads that mapped were excluded from downstream analysis. All non-human reads were mapped to HLI’s reference genome database. This database consists of a collection of ~11,900 genomes of bacteria, archaea, viruses, and eukaryotes downloaded from NCBI; the collection includes both complete as well as draft genomes. After read-mapping, an in-house implementation of an Expectation Maximization (EM) algorithm, similar to the GRAMMy algorithm^29^ was used to process the reads that were ambiguously mapped to multiple genomes in order to estimate Relative Genome Abundances (RGA). Based on the EM program’s assignment of reads to genomes, the genome coverage, which is the total length of mapped reads divided by the reference genome length, was calculated for each reference genome. The relative abundance of a reference genome is the genome coverage divided by the sum of all genome coverages. The relative abundances were aggregated at each taxonomic rank: species, genus, family, order, class and phylum. As a measure of confidence in unique taxonomic assignment, relative species abundances below the threshold of 10^−4^ were removed from further analysis. Non-human reads were assembled using IDBA-UD^30^ to generate contigs. Since the size of assembly results highly depends on the amount of input reads, we started with an equal amount of 6.77 million high quality reads for each sample for assembly. After human reads removal, the non-human reads used for assembly are 6.73–6.77 million.

### TIGRFAM/Pfam analysis

ORFs were predicted from scaffolds using MetaGene and compared with several reference protein or domain families, including COG, KOG, Pfam, TIGRFAM, and a comprehensive protein sequence database with RPS-BLAST (COG and KOG), Hmmer3 (Pfam and TIGRFAM) and NCBI BLASTP + (protein db). Here, the protein database contains the non-redundant proteins at 90% sequence identity from all proteins from the ~11,900 reference genomes. For metagenome samples, a portion of the reads, sometimes a significant portion of the reads, may not be assembled into scaffolds. These singleton reads were also analyzed and compared with the reference databases. For each sample, 5 subsets were made by randomly selecting reads from the singletons reads. Each subset is either 10% of the singleton reads or 100,000 reads, whichever is smaller. ORFs were predicted from these subsets using MetaGene and compared with the reference databases. The depth of coverage of scaffolds, based on the reads to scaffold mapping, were assigned to all the ORFs predicted from them. The depth of coverage of ORFs from a singleton reads subset is calculated as the total number of singleton reads divided by the number of reads in the subset.

Only the non-overlapping, top-scored alignments were used to calculate the protein family abundance, in a similar way of calculating the relative reference genome abundance. The protein family coverage is the sum of the alignment length multiplied by the depth of coverage of an ORF that hit the protein family, divided by the sequence length of the protein family. Both ORFs from the scaffolds and from singleton reads were considered. The contribution from the singletons was averaged from the 5 subsets. The relative protein family abundance is the protein family coverage divided by the sum of coverage of all protein families.

### Richness and Biodiversity Calculation and Normalization

For each sample taxonomic identities and relative abundance were assigned as described above (see Methods: Taxonomic assignment and assembly analysis). In addition to the threshold requirement of relative species abundance of at least 10^−4^, species with less than 100 read counts were also removed from richness and biodiversity analysis. Including only the species that met these threshold requirements, richness and biodiversity were calculated. Richness was calculated by counting the distinct number of individual species identified. Biodiversity was defined as Shannon’s Diversity Index (SDI)^31^, which accounts for the number of distinct species and the relative species abundances. Each sample was then normalized to the corresponding Basal sample from the same subject to enable comparisons across different subjects who can have different levels of diversity. These normalized scores did not appear to be a normal distribution as shown by visual inspection.

### Statistical analysis

#### Sequencing metrics

To determine significant differences in sequencing assembly metrics among the three sample types we used the Wilcoxon Ranked Sum test from the R statistical package^32^. Pair-wise comparisons of sequencing assembly metrics from all sample types were done using all samples (Basal (n = 16), Frozen (n = 48), and Stabilized (n = 48)). In some cases, comparisons were made between Day 0 Frozen (n = 16), Stabilized (n = 16), and Basal (n = 16) sample types. Comparisons were also made between Day 28 Frozen (n = 16), Stabilized (n = 16), and Basal (n = 16) sample types. The null hypothesis is that the distributions of a pair of sequencing assembly metrics are the same. The two-sided alternative hypothesis is that a difference is observed between the distributions of a pair of sequencing assembly metrics.

#### Nucleic acid yield analysis

Boxplots were generated at each timepoint for each treatment (Basal (n = 16); F0 (n = 16), F1 (n = 16), F28 (n = 16); S0 (n = 16), S1 (n = 16), S28 (n = 16)), using the boxplot function in R version 3.2.3. To determine significant differences between Stabilized nucleic acid yields and either Frozen or Basal yields, we used the Wilcoxon Ranked Sum test from the R statistical package^32^.

#### Precision Analysis

To assess the reproducibility between relative species abundances across timepoints within a Subject treatment (Frozen or Stabilized), Day 1 and Day 28 relative species abundance values were plotted against Day 0 relative species abundance values from the same treatment and Subject. Linear regression analysis was then used to determine the best fit linear regression line and to calculate r^2^- the goodness of fit of that line to the data. Results are listed in Table 2.

#### Diversity metrics

Statistical analysis of diversity was done using the Wilcoxon Rank Sum (ranksum), and ANOVA (f_oneway) tests found in the stats package of the SciPy library version 0.17^33^ in python. To assess if either Frozen or Stabilized samples change significantly over time, a one-way ANOVA test was used to compare each normalized Frozen or Stabilized time point within a given subject, across all subjects (F0 (n = 16), F1 (n = 16), F28 (n = 16); S0 (n = 16), S1 (n = 16), S28 (n = 16)). To assess if a significant difference between Basal (n = 16) samples and either Frozen (n = 48) or Stabilized (n = 48) samples exists, the distribution of Frozen/Stabilized samples was compared to the calculated percentile ranking of the Basal sample relative to the corresponding distribution using Wilcoxon Rank Sum.

## Additional Information

**How to cite this article**: Anderson, E. L. *et al*. A robust ambient temperature collection and stabilization strategy: Enabling worldwide functional studies of the human microbiome. *Sci. Rep.* **6**, 31731; doi: 10.1038/srep31731 (2016).

## Supplementary Material

Supplementary Information

## Figures and Tables

**Table 1:** Average QC and sequencing metrics.

Sample	Number passed reads (pair)	Fraction of Low quality reads	%Duplicated reads	Number mapped reads	Number unmapped reads	Number of non-human reads (pair)	% human reads (pair)	% Bacteria reads	% Archaea reads	% Eukaryota reads	% Virus reads	Total contig length (bp)	Contig N50 (bp)	Longest contig (bp)
Avg B	2.20E + 07	0.19	11.18	1.83E + 07	3.69E + 06	2.20E + 07	0.06	82.94	0.03	0.01	0.003	1.64E + 08	4192	5.06E + 05
Avg F	2.13E + 07	0.22	13.18	1.75E + 07	3.79E + 06	2.13E + 07	0.06	81.72	0.12	0.01	0.004	1.69E + 08	3792	4.58E + 05
Avg S	2.05E + 07	0.19	9.81	1.71E + 07	3.32E + 06	2.05E + 07	0.04	83.01	0.02	0.01	0.002	1.67E + 08	3816	5.03E + 05
Avg B0	2.27E + 07	0.20	11.52	1.89E + 07	3.83E + 06	2.27E + 07	0.06	82.86	0.03	0.01	0.003	1.67E + 08	3690	5.11E + 05
Avg F0	2.23E + 07	0.20	11.62	1.87E + 07	3.65E + 06	2.23E + 07	0.06	83.36	0.02	0.01	0.003	1.62E + 08	4397	4.81E + 05
Avg F1	1.99E + 07	0.21	12.82	1.66E + 07	3.30E + 06	1.98E + 07	0.06	82.87	0.07	0.01	0.003	1.56E + 08	4190	4.58E + 05
Avg F28	2.16E + 07	0.26	15.10	1.72E + 07	4.42E + 06	2.16E + 07	0.07	78.93	0.26	0.02	0.005	1.89E + 08	2791	4.35E + 05
Avg S0	2.07E + 07	0.19	9.85	1.73E + 07	3.38E + 06	2.07E + 07	0.04	82.75	0.02	0.01	0.002	1.67E + 08	4016	5.04E + 05
Avg S1	2.13E + 07	0.19	9.91	1.78E + 07	3.47E + 06	2.13E + 07	0.04	83.01	0.02	0.01	0.002	1.72E + 08	3932	5.08E + 05
Avg S28	1.94E + 07	0.20	9.66	1.63E + 07	3.11E + 06	1.94E + 07	0.04	83.27	0.01	0.01	0.002	1.62E + 08	3500	4.96E + 05

Average Sequencing Metrics. Sequencing metrics were tabulated for each sample type (Basal, Frozen, Stabilized) and values were averaged. Individual metrics are shown in Supplemental Table S2.

**Table 2:** Linear Regression of Subject relative species abundances on Day 0 compared to Day 1 and Day 28.

Subject	Timepoint	Frozen R^2^	Stabilized R^2^
1	Day 1	0.59526	0.98576
	Day 28	0.95473	0.95334
2	Day 1	0.96756	0.99855
	Day 28	0.95922	0.99443
3	Day 1	0.9683	0.99266
	Day 28	0.94875	0.97774
4	Day 1	0.97748	0.9855
	Day 28	0.97092	0.85906
5	Day 1	0.9002	0.9985
	Day 28	0.99002	0.9392
6	Day 1	0.99396	0.99433
	Day 28	0.9906	0.98294
7	Day 1	0.97744	0.99672
	Day 28	0.8606	0.93763
8	Day 1	0.99628	0.99118
	Day 28	0.99803	0.97414
9	Day 1	0.99524	0.99748
	Day 28	0.99457	0.99118
10	Day 1	0.9885	0.99808
	Day 28	0.98941	0.95439
11	Day 1	0.94093	0.99736
	Day 28	0.99289	0.99349
12	Day 1	0.78279	0.99447
	Day 28	0.97294	0.93879
13	Day 1	0.99896	0.9927
	Day 28	0.94033	0.91505
14	Day 1	0.84937	0.99611
	Day 28	0.92096	0.94392
15	Day 1	0.95923	0.99687
	Day 28	0.92947	0.97421
16	Day 1	0.97932	0.99223
	Day 28	0.96873	0.96901

Linear Regression of Precision Analysis. Linear regression lines (best-fitting straight lines that minimize the sum of squared errors of prediction) and r^2^ values (a measure of the regression lines goodness of fit) are listed for each subject, comparing Day 0 relative species abundance with the respective Day 1 or Day 28 relative species abundances, in both Frozen and Stabilized samples.
