# Supplementary material for: A robust ambient temperature collection and stabilization strategy: Enabling worldwide functional studies of the human microbiome
Source: Sci Rep. 2016 Aug 25;6:31731. doi: 10.1038/srep31731 (PMC4997331; doi:10.1038/srep31731)
Supplement: Supplementary Information [file srep31731-s1.pdf]

# A robust ambient temperature collection and stabilization strategy: Enabling worldwide functional studies of the human microbiome

Ericka L. Anderson[1*], Weizhong Li[1,2], Niels Klitgord[1], Sarah K. Highlander[2], Mark Dayrit[1], Victor Seguritan[1], Shibu Yooseph[1,2], William Biggs[1], J. Craig Venter[1,2], Karen E. Nelson[1,2], Marcus B. Jones[1]

[1] Human Longevity, Inc., San Diego, CA 92121.
[2] Genomic Medicine, J. Craig Venter Institute, La Jolla, CA 92037.
[*] Corresponding Author, eanderson@humanlongevity.com

Supplemental Information

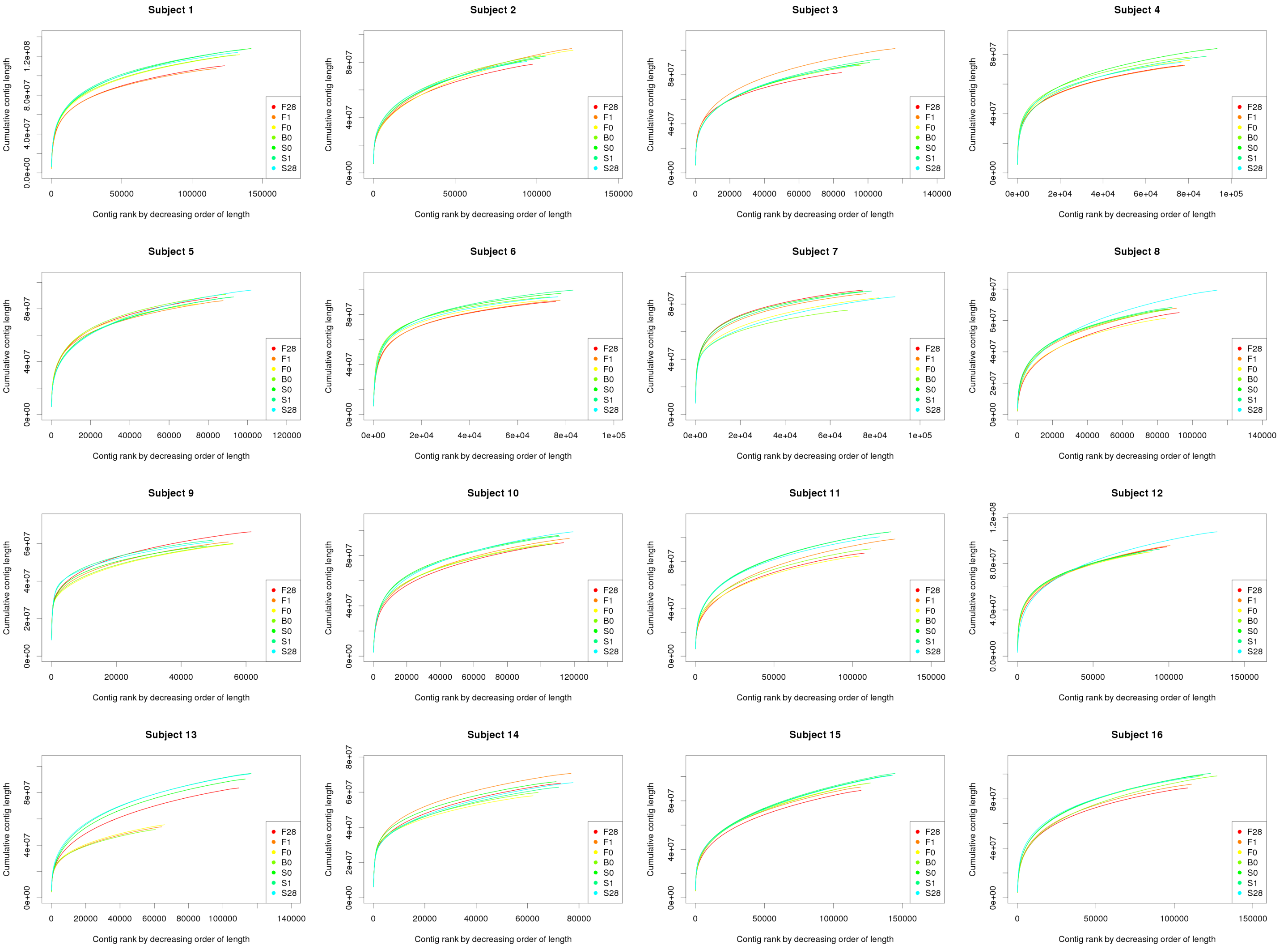


Supplemental Figure S1. Assembly Metrics are unaffected by stabilization method. Total contig length is plotted against contig rank in the order of decreasing length for all individual subjects. B0: Basal; F0, F1, F28: Frozen Day 0, Day 1, and Day 28; S0, S1, S28: Stabilized Day 0, Day 1, and Day 28.

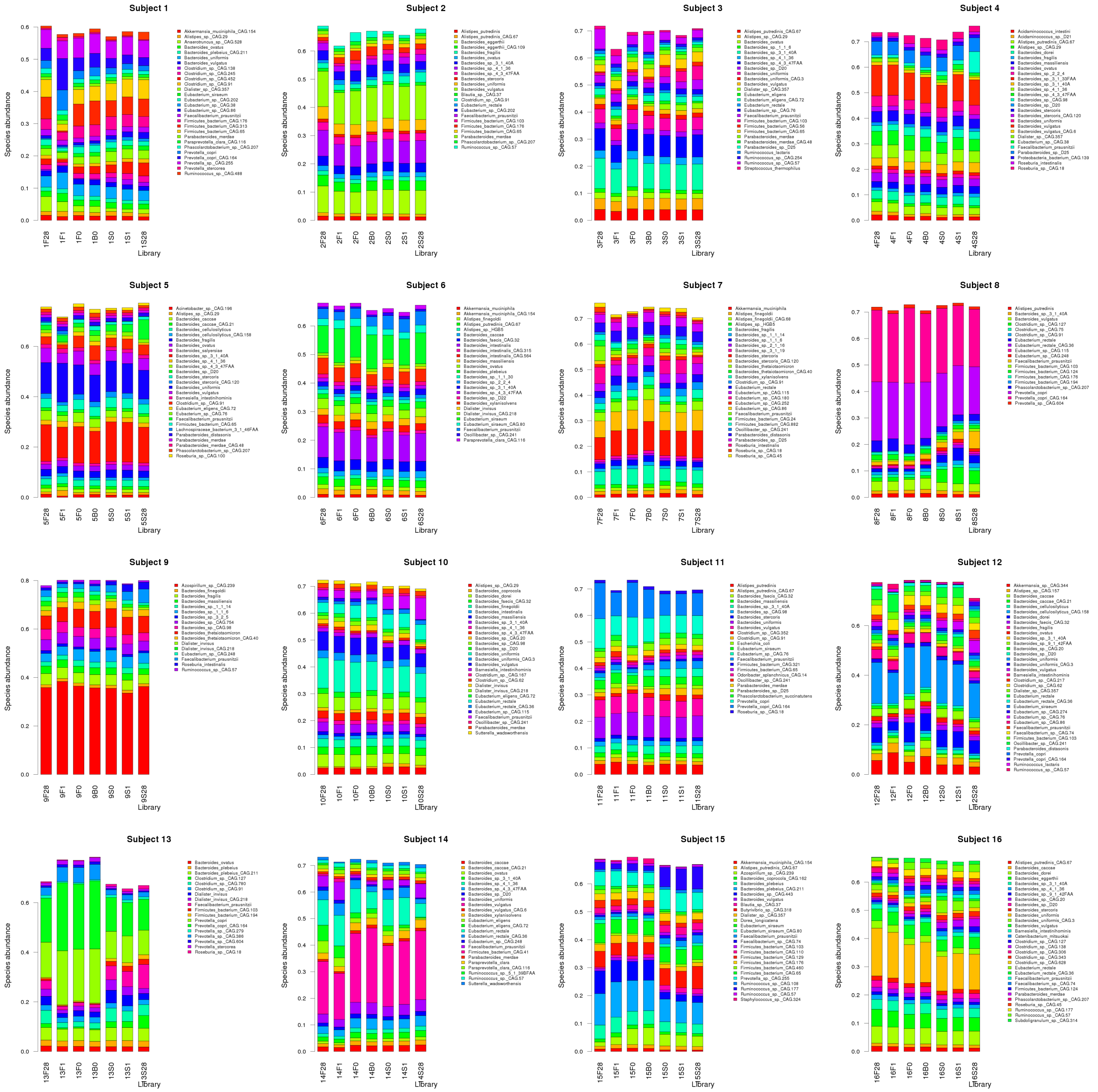


Supplemental Figure S2. Relative species abundance of individual subjects. Species abundance is plotted for Frozen, Basal, and Stabilized samples from each individual subject. B0: Basal; F0, F1, F28: Frozen Day 0, Day 1, and Day 28; S0, S1, S28: Stabilized Day 0, Day 1, and Day 28.

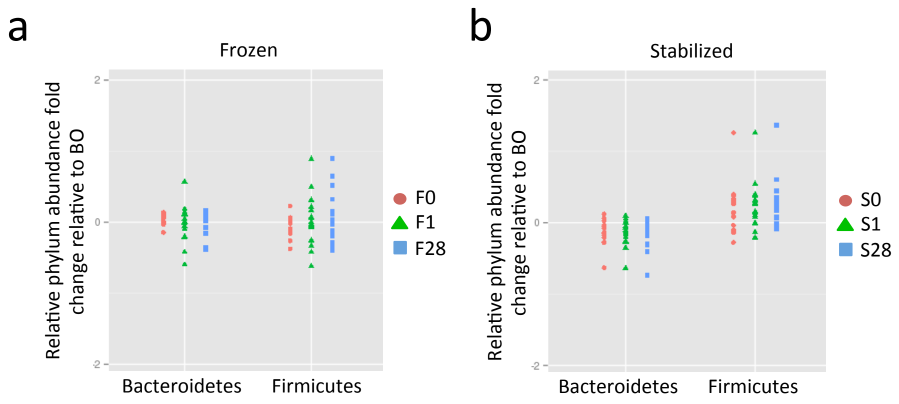


Supplemental Figure S3. Bacteroidetes and Firmicutes phlya are unchanged relative to Basal in Frozen and Stabilized samples. Fold change in Bacteroidetes or Firmicutes phyla relative to Basal across the time course in (a) Frozen or (b) Stabilized samples. F0, F1, F28: Frozen Day 0, Day 1, and Day 28; S0, S1, S28: Stabilized Day 0, Day 1, and Day 28.

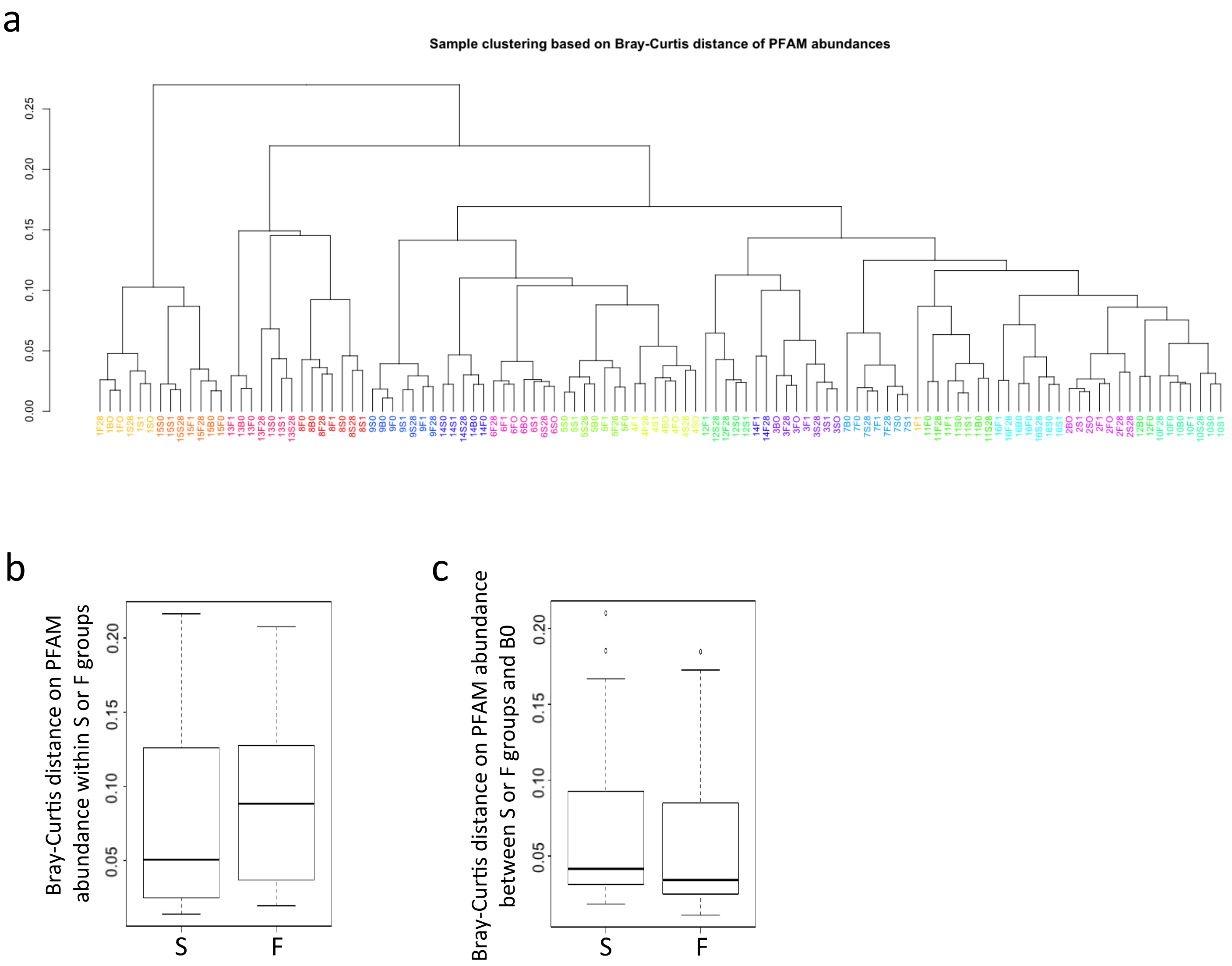


Supplemental Figure S4. Stabilized samples maintain results of Basal and Frozen Pfam analysis. (a) Bray-Curtis dissimilarity based on relative Pfam abundances were used to cluster all samples. Each color represents a distinct subject. (b) Box and whisker plots of calculated pairwise Bray-Curtis dissimilarity within stabilization method. (c) Box and whisker plots of calculated pairwise Bray-Curtis dissimilarity between Stabilized or Frozen samples and Basal. B0: Basal; F0, F1, F28: Frozen Day 0, Day 1, and Day 28; S0, S1, S28: Stabilized Day 0, Day 1, and Day 28; F: Frozen; S: Stabilized.

| Supplemental Table S1. Sequencing metrics from RNA seq on a Stabilized stool sample | | |
|---|---|---|
| **Sample** | **Stabilized RNA1A** | **Stabilized RNA1B** |
| **% rRNA** | 0.0165 | 0.0665 |
| **total rRNA** | 92743 | 255013 |
| **# 23S reads** | 62656 | 174727 |
| **# 16S reads** | 28573 | 79021 |
| **# 5S reads** | 1514 | 1265 |
| **# unmapped reads** | 807651 | 589168 |
| **# Viruses reads** | 656 | 859 |
| **# Eukaryota reads** | 903 | 1341 |
| **# Archaea reads** | 83 | 56 |
| **# Bacteria reads** | 4824571 | 3240490 |
| **# mapped reads** | 4826194 | 3242791 |
| **# non-human reads (pair)** | 5633845 | 3831959 |
| **# human reads (pair)** | 9168 | 13895 |
| **# of Low quality reads** | 0.2722 | 0.361 |
| **# good reads (pair)** | 5643013 | 3845854 |
| **Raw reads (GB)** | 1.938483 | 1.50487 |
| **# raw reads (pair)** | 7753933 | 6019481 |

**Sequencing metrics from an RNASeq Stabilized sample.**
Sequencing metrics were tabulated for sequencing libraries made from two replicate RNA extractions from a single Stabilized sample.

| Supplemental Table S2. Individual Sample QC and sequencing metrics | | | | | | | | | | | | | | |
|---|---|---|---|---|---|---|---|---|---|---|---|---|---|---|
| Sample | Number passed reads (pair) | Fraction of Low quality reads | %Duplicated reads | Number mapped reads | Number unmapped reads | Number of non-human reads (pair) | % human reads (pair) | % Bacteria reads | % Archaea reads | % Eukaryota reads | % Virus reads | Total contig length (bp) | Contig N50 (bp) | Longest contig (bp) |
| **1B0** | 1.7E+07 | 0.1517 | 15.87 | 1.3E+07 | 4.0E+06 | 1.7E+07 | 0.26 | 76.15 | 0.154 | 0.04 | 0.007 | 2.09E+08 | 4280 | 5.72E+05 |
| **2B0** | 2.3E+07 | 0.147 | 9.50 | 1.9E+07 | 3.9E+06 | 2.3E+07 | 0.04 | 82.60 | 0.002 | 0.00 | 0.007 | 1.91E+08 | 4073 | 3.27E+05 |
| **3B0** | 2.7E+07 | 0.2004 | 11.72 | 2.4E+07 | 3.7E+06 | 2.7E+07 | 0.01 | 86.64 | 0.002 | 0.01 | 0.001 | 1.96E+08 | 3630 | 3.61E+05 |
| **4B0** | 2.3E+07 | 0.1594 | 9.61 | 2.2E+07 | 1.9E+06 | 2.3E+07 | 0.05 | 91.72 | 0.000 | 0.00 | 0.002 | 1.51E+08 | 3382 | 8.13E+05 |
| **5B0** | 2.4E+07 | 0.1856 | 10.63 | 2.1E+07 | 3.0E+06 | 2.4E+07 | 0.01 | 87.32 | 0.001 | 0.00 | 0.001 | 1.70E+08 | 5651 | 4.44E+05 |
| **6B0** | 1.7E+07 | 0.1588 | 11.29 | 1.5E+07 | 2.4E+06 | 1.7E+07 | 0.02 | 86.19 | 0.020 | 0.00 | 0.007 | 1.38E+08 | 11349 | 4.10E+05 |
| **7B0** | 2.2E+07 | 0.1645 | 10.43 | 1.9E+07 | 3.1E+06 | 2.2E+07 | 0.08 | 85.76 | 0.001 | 0.00 | 0.002 | 1.34E+08 | 8773 | 1.09E+06 |
| **8B0** | 1.9E+07 | 0.1682 | 9.97 | 1.4E+07 | 4.6E+06 | 1.9E+07 | 0.03 | 75.16 | 0.025 | 0.00 | 0.003 | 1.42E+08 | 2191 | 2.54E+05 |
| **9B0** | 2.5E+07 | 0.2145 | 12.92 | 2.1E+07 | 3.9E+06 | 2.5E+07 | 0.07 | 84.06 | 0.001 | 0.00 | 0.000 | 1.17E+08 | 4527 | 8.02E+05 |
| **10B0** | 2.3E+07 | 0.189 | 10.22 | 2.0E+07 | 3.2E+06 | 2.3E+07 | 0.02 | 86.20 | 0.001 | 0.05 | 0.001 | 1.80E+08 | 2580 | 4.74E+05 |
| **11B0** | 2.1E+07 | 0.2239 | 15.56 | 1.7E+07 | 4.1E+06 | 2.1E+07 | 0.09 | 80.11 | 0.001 | 0.01 | 0.002 | 1.70E+08 | 2654 | 3.28E+05 |
| **12B0** | 1.9E+07 | 0.2228 | 10.28 | 1.7E+07 | 2.5E+06 | 1.9E+07 | 0.06 | 86.91 | 0.001 | 0.00 | 0.000 | 1.49E+08 | 4770 | 4.13E+05 |
| **13B0** | 2.1E+07 | 0.2581 | 11.28 | 1.6E+07 | 5.2E+06 | 2.1E+07 | 0.12 | 74.70 | 0.002 | 0.01 | 0.000 | 1.11E+08 | 1860 | 3.07E+05 |
| **14B0** | 2.2E+07 | 0.2194 | 8.86 | 1.9E+07 | 3.1E+06 | 2.2E+07 | 0.03 | 85.66 | 0.000 | 0.01 | 0.006 | 1.20E+08 | 2457 | 4.65E+05 |
| **15B0** | 2.7E+07 | 0.2129 | 9.32 | 2.0E+07 | 6.4E+06 | 2.7E+07 | 0.07 | 75.59 | 0.076 | 0.03 | 0.007 | 2.53E+08 | 1438 | 4.16E+05 |
| **16B0** | 2.3E+07 | 0.2159 | 11.44 | 1.9E+07 | 4.1E+06 | 2.3E+07 | 0.02 | 82.22 | 0.121 | 0.01 | 0.003 | 1.92E+08 | 3450 | 6.28E+05 |
| **1F0** | 1.7E+07 | 0.1577 | 17.52 | 1.3E+07 | 4.1E+06 | 1.7E+07 | 0.28 | 75.77 | 0.153 | 0.04 | 0.007 | 2.10E+08 | 3991 | 4.61E+05 |
| **2F0** | 3.2E+07 | 0.2187 | 11.16 | 2.6E+07 | 5.7E+06 | 3.2E+07 | 0.11 | 82.24 | 0.002 | 0.00 | 0.007 | 2.10E+08 | 3991 | 4.61E+05 |
| **3F0** | 2.7E+07 | 0.2046 | 11.95 | 2.4E+07 | 3.4E+06 | 2.7E+07 | 0.01 | 87.33 | 0.002 | 0.01 | 0.001 | 2.39E+08 | 2923 | 3.04E+05 |
| **4F0** | 2.4E+07 | 0.1673 | 10.29 | 2.2E+07 | 1.9E+06 | 2.4E+07 | 0.03 | 92.13 | 0.000 | 0.00 | 0.002 | 1.91E+08 | 4055 | 4.66E+05 |
| **5F0** | 2.1E+07 | 0.1714 | 11.67 | 1.8E+07 | 2.3E+06 | 2.1E+07 | 0.02 | 89.04 | 0.001 | 0.00 | 0.001 | 1.47E+08 | 3131 | 8.18E+05 |
| **6F0** | 1.7E+07 | 0.1705 | 13.38 | 1.5E+07 | 2.3E+06 | 1.7E+07 | 0.04 | 86.89 | 0.020 | 0.00 | 0.005 | 1.48E+08 | 6546 | 4.44E+05 |
| **7F0** | 2.5E+07 | 0.1786 | 9.93 | 2.1E+07 | 3.8E+06 | 2.5E+07 | 0.07 | 84.41 | 0.001 | 0.00 | 0.002 | 1.30E+08 | 12205 | 4.09E+05 |
| **8F0** | 1.9E+07 | 0.1917 | 12.97 | 1.5E+07 | 4.5E+06 | 1.9E+07 | 0.03 | 76.15 | 0.014 | 0.00 | 0.002 | 1.56E+08 | 8306 | 8.94E+05 |
| **9F0** | 2.5E+07 | 0.2031 | 9.50 | 2.1E+07 | 3.8E+06 | 2.5E+07 | 0.07 | 84.40 | 0.001 | 0.00 | 0.000 | 1.28E+08 | 2393 | 2.18E+05 |
| **10F0** | 2.4E+07 | 0.1865 | 10.21 | 2.1E+07 | 3.2E+06 | 2.4E+07 | 0.03 | 86.72 | 0.001 | 0.05 | 0.001 | 1.17E+08 | 6024 | 8.01E+05 |
| **11F0** | 2.2E+07 | 0.2278 | 17.76 | 1.8E+07 | 4.1E+06 | 2.2E+07 | 0.05 | 81.51 | 0.001 | 0.01 | 0.001 | 1.85E+08 | 2823 | 5.48E+05 |
| **12F0** | 2.1E+07 | 0.2453 | 10.58 | 1.8E+07 | 2.6E+06 | 2.1E+07 | 0.03 | 87.39 | 0.001 | 0.00 | 0.000 | 1.59E+08 | 2996 | 3.28E+05 |

## Supplemental Table S2 cont'd

| | | | | | | | | | | | | | | |
|---|---|---|---|---|---|---|---|---|---|---|---|---|---|---|
| **13F0** | 2.0E+07 | 0.2643 | 11.31 | 1.5E+07 | 5.0E+06 | 2.0E+07 | 0.03 | 75.17 | 0.002 | 0.00 | 0.000 | 1.54E+08 | 4684 | 3.91E+05 |
| **14F0** | 2.1E+07 | 0.2129 | 8.84 | 1.8E+07 | 2.7E+06 | 2.1E+07 | 0.02 | 86.88 | 0.001 | 0.01 | 0.006 | 1.13E+08 | 2075 | 3.07E+05 |
| **15F0** | 2.4E+07 | 0.2081 | 8.34 | 1.8E+07 | 5.8E+06 | 2.4E+07 | 0.07 | 75.99 | 0.060 | 0.02 | 0.007 | 1.11E+08 | 2790 | 4.65E+05 |
| **16F0** | 1.9E+07 | 0.191 | 10.54 | 1.6E+07 | 3.5E+06 | 1.9E+07 | 0.03 | 81.73 | 0.135 | 0.01 | 0.005 | 2.35E+08 | 1605 | 3.83E+05 |
| **1F1** | 1.5E+07 | 0.1733 | 24.34 | 1.3E+07 | 2.9E+06 | 1.5E+07 | 0.23 | 80.39 | 0.499 | 0.01 | 0.002 | 1.67E+08 | 3794 | 3.98E+05 |
| **2F1** | 2.1E+07 | 0.173 | 14.65 | 1.7E+07 | 3.6E+06 | 2.1E+07 | 0.03 | 82.56 | 0.002 | 0.01 | 0.010 | 1.90E+08 | 3364 | 3.46E+05 |
| **3F1** | 2.0E+07 | 0.1842 | 14.40 | 1.7E+07 | 2.9E+06 | 2.0E+07 | 0.02 | 85.09 | 0.002 | 0.01 | 0.002 | 1.95E+08 | 3944 | 4.17E+05 |
| **4F1** | 2.1E+07 | 0.1721 | 9.64 | 1.9E+07 | 1.6E+06 | 2.1E+07 | 0.02 | 92.33 | 0.000 | 0.00 | 0.001 | 1.33E+08 | 2889 | 8.13E+05 |
| **5F1** | 2.1E+07 | 0.2226 | 12.71 | 1.8E+07 | 3.1E+06 | 2.1E+07 | 0.02 | 85.32 | 0.001 | 0.00 | 0.003 | 1.64E+08 | 3911 | 4.73E+05 |
| **6F1** | 1.8E+07 | 0.1906 | 16.11 | 1.5E+07 | 2.3E+06 | 1.8E+07 | 0.07 | 87.05 | 0.041 | 0.00 | 0.008 | 1.31E+08 | 10404 | 4.09E+05 |
| **7F1** | 2.0E+07 | 0.1637 | 12.48 | 1.7E+07 | 3.1E+06 | 2.0E+07 | 0.05 | 84.40 | 0.001 | 0.00 | 0.002 | 1.42E+08 | 12646 | 8.94E+05 |
| **8F1** | 2.1E+07 | 0.1979 | 12.78 | 1.6E+07 | 4.9E+06 | 2.1E+07 | 0.06 | 76.42 | 0.100 | 0.00 | 0.003 | 1.49E+08 | 2357 | 3.24E+05 |
| **9F1** | 1.9E+07 | 0.1993 | 8.09 | 1.6E+07 | 3.2E+06 | 1.9E+07 | 0.15 | 83.36 | 0.001 | 0.00 | 0.000 | 1.07E+08 | 5507 | 5.73E+05 |
| **10F1** | 2.9E+07 | 0.2074 | 11.88 | 2.5E+07 | 4.1E+06 | 2.9E+07 | 0.02 | 85.92 | 0.001 | 0.04 | 0.001 | 2.01E+08 | 2828 | 5.47E+05 |
| **11F1** | 2.3E+07 | 0.2821 | 14.69 | 1.8E+07 | 4.4E+06 | 2.3E+07 | 0.10 | 80.79 | 0.002 | 0.01 | 0.002 | 1.92E+08 | 2637 | 3.63E+05 |
| **12F1** | 1.8E+07 | 0.2687 | 14.66 | 1.5E+07 | 3.1E+06 | 1.8E+07 | 0.04 | 82.82 | 0.002 | 0.00 | 0.000 | 1.55E+08 | 4169 | 4.24E+05 |
| **13F1** | 7.3E+06 | 0.2563 | 7.00 | 5.4E+06 | 1.8E+06 | 7.3E+06 | 0.03 | 74.95 | 0.002 | 0.01 | 0.000 | 5.69E+07 | 1560 | 2.24E+05 |
| **14F1** | 2.4E+07 | 0.2817 | 9.61 | 2.0E+07 | 3.7E+06 | 2.3E+07 | 0.03 | 84.22 | 0.001 | 0.01 | 0.005 | 1.44E+08 | 2017 | 4.64E+05 |
| **15F1** | 2.2E+07 | 0.1956 | 8.80 | 1.7E+07 | 5.0E+06 | 2.2E+07 | 0.06 | 76.82 | 0.190 | 0.02 | 0.006 | 2.16E+08 | 1550 | 3.73E+05 |
| **16F1** | 1.8E+07 | 0.196 | 13.21 | 1.5E+07 | 3.0E+06 | 1.8E+07 | 0.02 | 83.52 | 0.308 | 0.01 | 0.004 | 1.57E+08 | 3459 | 2.89E+05 |
| **1F28** | 1.4E+07 | 0.1694 | 26.87 | 1.1E+07 | 2.8E+06 | 1.4E+07 | 0.11 | 79.60 | 0.066 | 0.01 | 0.001 | 1.45E+08 | 2792 | 4.51E+05 |
| **2F28** | 2.3E+07 | 0.1955 | 18.23 | 1.9E+07 | 4.6E+06 | 2.3E+07 | 0.03 | 79.96 | 0.003 | 0.01 | 0.013 | 1.69E+08 | 3224 | 2.71E+05 |
| **3F28** | 2.5E+07 | 0.2418 | 17.85 | 2.1E+07 | 3.9E+06 | 2.5E+07 | 0.02 | 84.15 | 0.002 | 0.01 | 0.002 | 1.63E+08 | 3831 | 4.22E+05 |
| **4F28** | 2.8E+07 | 0.2299 | 11.54 | 2.5E+07 | 2.9E+06 | 2.8E+07 | 0.01 | 89.58 | 0.001 | 0.00 | 0.003 | 1.42E+08 | 2939 | 8.18E+05 |
| **5F28** | 2.6E+07 | 0.299 | 14.14 | 2.3E+07 | 3.8E+06 | 2.6E+07 | 0.01 | 85.76 | 0.002 | 0.00 | 0.001 | 1.56E+08 | 6474 | 4.16E+05 |
| **6F28** | 2.2E+07 | 0.2164 | 13.06 | 1.7E+07 | 5.1E+06 | 2.2E+07 | 0.05 | 76.72 | 0.337 | 0.00 | 0.019 | 1.40E+08 | 13134 | 4.40E+05 |
| **7F28** | 2.4E+07 | 0.2753 | 10.59 | 1.8E+07 | 5.8E+06 | 2.4E+07 | 0.12 | 75.61 | 0.003 | 0.01 | 0.007 | 1.49E+08 | 8002 | 1.09E+06 |
| **8F28** | 2.3E+07 | 0.267 | 10.63 | 1.7E+07 | 5.7E+06 | 2.3E+07 | 0.05 | 74.79 | 0.480 | 0.01 | 0.009 | 1.64E+08 | 1576 | 2.77E+05 |
| **9F28** | 2.4E+07 | 0.2435 | 10.30 | 2.0E+07 | 4.4E+06 | 2.4E+07 | 0.08 | 81.51 | 0.002 | 0.00 | 0.000 | 1.26E+08 | 4557 | 8.02E+05 |
| **10F28** | 2.2E+07 | 0.3223 | 11.62 | 1.7E+07 | 4.5E+06 | 2.2E+07 | 0.05 | 79.37 | 0.003 | 0.24 | 0.005 | 1.85E+08 | 2023 | 5.38E+05 |
| **11F28** | 1.8E+07 | 0.3082 | 30.59 | 1.3E+07 | 4.6E+06 | 1.8E+07 | 0.24 | 74.51 | 0.003 | 0.01 | 0.004 | 1.42E+08 | 3104 | 4.36E+05 |
| **12F28** | 1.6E+07 | 0.2724 | 13.66 | 1.3E+07 | 3.5E+06 | 1.6E+07 | 0.07 | 78.75 | 0.002 | 0.01 | 0.000 | 1.56E+08 | 4197 | 3.91E+05 |

# Supplemental Table S2 cont'd

| | | | | | | | | | | | | | | |
|---|---|---|---|---|---|---|---|---|---|---|---|---|---|---|
| **13F28** | 2.2E+07 | 0.3375 | 13.53 | 1.6E+07 | 5.2E+06 | 2.2E+07 | 0.11 | 75.93 | 0.003 | 0.01 | 0.001 | 1.59E+08 | 2740 | 2.54E+05 |
| **14F28** | 2.0E+07 | 0.3413 | 12.04 | 1.6E+07 | 4.0E+06 | 2.0E+07 | 0.03 | 80.07 | 0.001 | 0.02 | 0.005 | 1.20E+08 | 2730 | 4.65E+05 |
| **15F28** | 2.2E+07 | 0.2542 | 9.01 | 1.5E+07 | 6.7E+06 | 2.2E+07 | 0.08 | 67.92 | 1.523 | 0.01 | 0.009 | 2.19E+08 | 1396 | 4.38E+05 |
| **16F28** | 1.6E+07 | 0.2201 | 17.99 | 1.3E+07 | 3.2E+06 | 1.6E+07 | 0.03 | 78.64 | 1.772 | 0.01 | 0.007 | 1.38E+08 | 2988 | 3.33E+05 |
| **1S0** | 1.5E+07 | 0.1442 | 13.79 | 1.2E+07 | 3.8E+06 | 1.5E+07 | 0.10 | 75.13 | 0.135 | 0.02 | 0.005 | 2.10E+08 | 4170 | 6.07E+05 |
| **2S0** | 2.0E+07 | 0.1542 | 10.95 | 1.6E+07 | 3.5E+06 | 2.0E+07 | 0.01 | 82.43 | 0.002 | 0.00 | 0.007 | 1.76E+08 | 3455 | 2.92E+05 |
| **3S0** | 2.8E+07 | 0.2153 | 10.84 | 2.4E+07 | 3.9E+06 | 2.8E+07 | 0.01 | 86.22 | 0.002 | 0.00 | 0.001 | 2.10E+08 | 2876 | 3.62E+05 |
| **4S0** | 2.9E+07 | 0.1718 | 9.34 | 2.6E+07 | 2.4E+06 | 2.9E+07 | 0.01 | 91.58 | 0.001 | 0.00 | 0.002 | 1.71E+08 | 3865 | 8.18E+05 |
| **5S0** | 2.7E+07 | 0.1806 | 11.40 | 2.4E+07 | 3.1E+06 | 2.7E+07 | 0.01 | 88.55 | 0.001 | 0.00 | 0.000 | 1.88E+08 | 3957 | 5.01E+05 |
| **6S0** | 1.6E+07 | 0.162 | 11.11 | 1.4E+07 | 2.2E+06 | 1.6E+07 | 0.05 | 86.85 | 0.012 | 0.00 | 0.004 | 1.36E+08 | 10756 | 3.38E+05 |
| **7S0** | 2.3E+07 | 0.1712 | 9.38 | 1.9E+07 | 3.8E+06 | 2.3E+07 | 0.01 | 83.69 | 0.002 | 0.00 | 0.001 | 1.58E+08 | 8139 | 1.09E+06 |
| **8S0** | 1.9E+07 | 0.1757 | 9.43 | 1.4E+07 | 4.4E+06 | 1.9E+07 | 0.03 | 76.43 | 0.015 | 0.00 | 0.002 | 1.41E+08 | 2039 | 3.24E+05 |
| **9S0** | 2.4E+07 | 0.1834 | 8.92 | 2.1E+07 | 3.7E+06 | 2.4E+07 | 0.08 | 84.44 | 0.001 | 0.00 | 0.000 | 1.11E+08 | 5678 | 8.02E+05 |
| **10S0** | 2.1E+07 | 0.1723 | 9.56 | 1.8E+07 | 3.1E+06 | 2.1E+07 | 0.03 | 85.16 | 0.002 | 0.02 | 0.001 | 1.80E+08 | 2864 | 5.38E+05 |
| **11S0** | 2.2E+07 | 0.2429 | 12.13 | 1.8E+07 | 4.0E+06 | 2.2E+07 | 0.11 | 81.71 | 0.002 | 0.01 | 0.001 | 1.92E+08 | 3168 | 3.56E+05 |
| **12S0** | 1.8E+07 | 0.2314 | 8.79 | 1.6E+07 | 2.7E+06 | 1.8E+07 | 0.07 | 85.08 | 0.002 | 0.01 | 0.000 | 1.52E+08 | 4315 | 4.23E+05 |
| **13S0** | 6.8E+06 | 0.2731 | 6.05 | 5.0E+06 | 1.8E+06 | 6.8E+06 | 0.03 | 74.02 | 0.002 | 0.01 | 0.000 | 9.04E+07 | 1240 | 3.07E+05 |
| **14S0** | 2.1E+07 | 0.2131 | 8.24 | 1.8E+07 | 3.1E+06 | 2.1E+07 | 0.02 | 85.24 | 0.001 | 0.01 | 0.004 | 1.28E+08 | 2629 | 4.65E+05 |
| **15S0** | 2.2E+07 | 0.1952 | 7.79 | 1.7E+07 | 5.4E+06 | 2.2E+07 | 0.03 | 75.50 | 0.043 | 0.01 | 0.005 | 2.46E+08 | 1647 | 3.86E+05 |
| **16S0** | 1.9E+07 | 0.1899 | 9.82 | 1.6E+07 | 3.4E+06 | 1.9E+07 | 0.02 | 82.03 | 0.099 | 0.01 | 0.003 | 1.81E+08 | 3451 | 4.50E+05 |
| **1S1** | 1.2E+07 | 0.1385 | 12.59 | 9.2E+06 | 2.9E+06 | 1.2E+07 | 0.09 | 75.85 | 0.098 | 0.02 | 0.005 | 1.81E+08 | 3932 | 4.91E+05 |
| **2S1** | 1.8E+07 | 0.1524 | 10.25 | 1.5E+07 | 3.0E+06 | 1.8E+07 | 0.01 | 82.79 | 0.002 | 0.00 | 0.007 | 1.69E+08 | 2928 | 2.94E+05 |
| **3S1** | 2.6E+07 | 0.198 | 10.80 | 2.3E+07 | 3.5E+06 | 2.6E+07 | 0.01 | 86.35 | 0.002 | 0.01 | 0.001 | 2.10E+08 | 3061 | 3.62E+05 |
| **4S1** | 2.9E+07 | 0.1857 | 8.97 | 2.7E+07 | 2.2E+06 | 2.9E+07 | 0.01 | 92.46 | 0.001 | 0.00 | 0.001 | 1.61E+08 | 3212 | 7.37E+05 |
| **5S1** | 2.7E+07 | 0.187 | 11.01 | 2.4E+07 | 3.1E+06 | 2.7E+07 | 0.01 | 88.50 | 0.001 | 0.00 | 0.000 | 1.90E+08 | 3597 | 4.31E+05 |
| **6S1** | 2.1E+07 | 0.1708 | 10.15 | 1.8E+07 | 2.8E+06 | 2.1E+07 | 0.05 | 86.36 | 0.019 | 0.00 | 0.006 | 1.52E+08 | 11826 | 3.38E+05 |
| **7S1** | 2.3E+07 | 0.1714 | 9.03 | 1.9E+07 | 3.5E+06 | 2.3E+07 | 0.01 | 84.31 | 0.002 | 0.00 | 0.001 | 1.58E+08 | 7339 | 1.09E+06 |
| **8S1** | 1.8E+07 | 0.1738 | 9.33 | 1.4E+07 | 4.2E+06 | 1.8E+07 | 0.03 | 77.32 | 0.011 | 0.00 | 0.002 | 1.43E+08 | 1995 | 3.24E+05 |
| **9S1** | 2.3E+07 | 0.1812 | 8.39 | 1.9E+07 | 3.5E+06 | 2.3E+07 | 0.08 | 84.34 | 0.001 | 0.00 | 0.000 | 1.12E+08 | 5251 | 8.03E+05 |
| **10S1** | 2.4E+07 | 0.1686 | 9.57 | 2.0E+07 | 3.5E+06 | 2.4E+07 | 0.03 | 85.28 | 0.002 | 0.02 | 0.000 | 1.94E+08 | 2789 | 6.62E+05 |
| **11S1** | 2.0E+07 | 0.2246 | 12.57 | 1.6E+07 | 3.7E+06 | 2.0E+07 | 0.11 | 81.34 | 0.001 | 0.01 | 0.001 | 1.84E+08 | 3081 | 3.28E+05 |
| **12S1** | 2.0E+07 | 0.2254 | 9.11 | 1.7E+07 | 3.0E+06 | 2.0E+07 | 0.03 | 84.99 | 0.002 | 0.00 | 0.000 | 1.62E+08 | 3965 | 3.90E+05 |

## Supplemental Table S2 cont'd

| | | | | | | | | | | | | | | |
|---|---|---|---|---|---|---|---|---|---|---|---|---|---|---|
| **13S1** | 2.0E+07 | 0.2549 | 10.98 | 1.5E+07 | 5.3E+06 | 2.0E+07 | 0.03 | 74.15 | 0.002 | 0.01 | 0.000 | 1.81E+08 | 2728 | 4.92E+05 |
| **14S1** | 2.1E+07 | 0.2205 | 8.33 | 1.8E+07 | 2.8E+06 | 2.1E+07 | 0.02 | 86.65 | 0.001 | 0.01 | 0.004 | 1.28E+08 | 2098 | 4.65E+05 |
| **15S1** | 2.1E+07 | 0.1952 | 7.82 | 1.6E+07 | 5.1E+06 | 2.1E+07 | 0.03 | 75.50 | 0.041 | 0.01 | 0.004 | 2.40E+08 | 1610 | 3.86E+05 |
| **16S1** | 1.9E+07 | 0.1819 | 9.65 | 1.6E+07 | 3.4E+06 | 1.9E+07 | 0.02 | 82.00 | 0.074 | 0.01 | 0.003 | 1.83E+08 | 3499 | 5.32E+05 |
| **1S28** | 8.6E+06 | 0.1372 | 12.91 | 6.6E+06 | 2.0E+06 | 8.6E+06 | 0.12 | 76.25 | 0.062 | 0.02 | 0.005 | 1.46E+08 | 2757 | 7.23E+05 |
| **2S28** | 1.2E+07 | 0.1412 | 8.84 | 9.6E+06 | 2.1E+06 | 1.2E+07 | 0.02 | 82.40 | 0.002 | 0.01 | 0.005 | 1.21E+08 | 2735 | 2.85E+05 |
| **3S28** | 2.4E+07 | 0.1934 | 11.93 | 2.1E+07 | 3.1E+06 | 2.4E+07 | 0.01 | 86.87 | 0.002 | 0.01 | 0.001 | 1.88E+08 | 3033 | 3.56E+05 |
| **4S28** | 2.7E+07 | 0.1732 | 9.82 | 2.5E+07 | 2.1E+06 | 2.7E+07 | 0.01 | 92.36 | 0.000 | 0.00 | 0.001 | 1.47E+08 | 3549 | 8.13E+05 |
| **5S28** | 2.5E+07 | 0.2105 | 9.27 | 2.2E+07 | 2.9E+06 | 2.5E+07 | 0.01 | 88.67 | 0.001 | 0.00 | 0.000 | 1.77E+08 | 4192 | 3.60E+05 |
| **6S28** | 1.6E+07 | 0.1607 | 10.79 | 1.4E+07 | 2.1E+06 | 1.6E+07 | 0.05 | 87.20 | 0.009 | 0.00 | 0.003 | 1.33E+08 | 10284 | 4.09E+05 |
| **7S28** | 2.5E+07 | 0.236 | 11.43 | 2.1E+07 | 3.8E+06 | 2.5E+07 | 0.04 | 84.80 | 0.001 | 0.00 | 0.001 | 1.59E+08 | 5468 | 1.09E+06 |
| **8S28** | 2.0E+07 | 0.1867 | 8.62 | 1.5E+07 | 4.7E+06 | 2.0E+07 | 0.03 | 76.69 | 0.017 | 0.00 | 0.002 | 1.70E+08 | 1564 | 3.24E+05 |
| **9S28** | 2.3E+07 | 0.203 | 8.43 | 2.0E+07 | 3.5E+06 | 2.3E+07 | 0.07 | 84.89 | 0.001 | 0.00 | 0.000 | 1.11E+08 | 5656 | 6.21E+05 |
| **10S28** | 2.2E+07 | 0.1936 | 8.69 | 1.9E+07 | 3.3E+06 | 2.2E+07 | 0.03 | 84.72 | 0.002 | 0.02 | 0.001 | 1.94E+08 | 2483 | 4.74E+05 |
| **11S28** | 1.5E+07 | 0.2154 | 10.64 | 1.2E+07 | 2.7E+06 | 1.5E+07 | 0.09 | 81.81 | 0.001 | 0.01 | 0.001 | 1.57E+08 | 2855 | 3.28E+05 |
| **12S28** | 1.6E+07 | 0.2223 | 7.97 | 1.4E+07 | 2.6E+06 | 1.6E+07 | 0.06 | 84.26 | 0.001 | 0.00 | 0.000 | 1.82E+08 | 2063 | 3.83E+05 |
| **13S28** | 1.9E+07 | 0.2697 | 8.99 | 1.4E+07 | 4.7E+06 | 1.9E+07 | 0.03 | 75.30 | 0.002 | 0.01 | 0.000 | 1.76E+08 | 2477 | 5.28E+05 |
| **14S28** | 2.0E+07 | 0.2212 | 8.47 | 1.8E+07 | 2.5E+06 | 2.0E+07 | 0.02 | 87.68 | 0.000 | 0.01 | 0.004 | 1.31E+08 | 1981 | 4.65E+05 |
| **15S28** | 2.2E+07 | 0.2004 | 7.61 | 1.7E+07 | 5.1E+06 | 2.2E+07 | 0.03 | 76.63 | 0.027 | 0.01 | 0.004 | 2.42E+08 | 1635 | 3.87E+05 |
| **16S28** | 1.5E+07 | 0.1871 | 10.18 | 1.2E+07 | 2.6E+06 | 1.5E+07 | 0.02 | 81.74 | 0.086 | 0.01 | 0.004 | 1.56E+08 | 3270 | 3.98E+05 |

**Individual Sample Sequencing Metrics.** Sequencing metrics were tabulated for each sample. Averaged sequencing metrics are shown in Table 1.

| Supplementary Table S3. Sequencing assembly metrics | | | | | | |
|---|---|---|---|---|---|---|
| **Sample** | **Treatment** | **Subject** | **Number non-human reads for assembly (pair)** | **Total contig length (bp)** | **Contig N50 (bp)** | **Longest contig (bp)** |
| **1B0** | B0 | 1 | 6,747,334 | 121,352,726 | 2,189 | 110,081 |
| **2B0** | B0 | 2 | 6,762,180 | 84,771,234 | 1,398 | 451,141 |
| **3B0** | B0 | 3 | 6,764,056 | 87,833,571 | 1,903 | 251,829 |
| **4B0** | B0 | 4 | 6,762,066 | 78,448,068 | 2,768 | 185,182 |
| **5B0** | B0 | 5 | 6,764,199 | 90,952,224 | 2,450 | 272,345 |
| **6B0** | B0 | 6 | 6,763,544 | 94,205,222 | 6,952 | 210,048 |
| **7B0** | B0 | 7 | 6,759,682 | 75,589,632 | 6,073 | 236,758 |
| **8B0** | B0 | 8 | 6,762,882 | 66,958,700 | 1,515 | 364,716 |
| **9B0** | B0 | 9 | 6,760,203 | 60,027,286 | 3,836 | 361,176 |
| **10B0** | B0 | 10 | 6,763,757 | 89,549,999 | 1,756 | 215,299 |
| **11B0** | B0 | 11 | 6,758,786 | 90,521,351 | 1,370 | 230,787 |
| **12B0** | B0 | 12 | 6,761,243 | 89,549,600 | 2,599 | 342,996 |
| **13B0** | B0 | 13 | 6,756,597 | 51,930,157 | 1,891 | 359,668 |
| **14B0** | B0 | 14 | 6,763,289 | 59,863,002 | 2,981 | 363,194 |
| **15B0** | B0 | 15 | 6,760,612 | 94,799,537 | 1,164 | 469,211 |
| **16B0** | B0 | 16 | 6,763,952 | 98,503,972 | 1,246 | 134,392 |
| **1F0** | F0 | 1 | 6,745,916 | 121,687,862 | 2,074 | 117,508 |
| **2F0** | F0 | 2 | 6,757,493 | 88,704,376 | 987 | 434,010 |
| **3F0** | F0 | 3 | 6,764,309 | 88,990,759 | 1,886 | 217,752 |
| **4F0** | F0 | 4 | 6,763,109 | 76,017,652 | 2,486 | 223,525 |
| **5F0** | F0 | 5 | 6,763,983 | 83,704,561 | 2,946 | 277,815 |
| **6F0** | F0 | 6 | 6,762,479 | 91,621,948 | 6,195 | 339,338 |
| **7F0** | F0 | 7 | 6,760,357 | 84,575,506 | 4,044 | 206,096 |
| **8F0** | F0 | 8 | 6,762,917 | 61,421,024 | 1,215 | 235,064 |
| **9F0** | F0 | 9 | 6,760,330 | 59,830,297 | 3,703 | 360,105 |
| **10F0** | F0 | 10 | 6,763,186 | 90,549,075 | 1,728 | 469,082 |
| **11F0** | F0 | 11 | 6,761,435 | 84,335,355 | 1,324 | 132,083 |
| **12F0** | F0 | 12 | 6,763,038 | 90,244,109 | 2,382 | 426,787 |
| **13F0** | F0 | 13 | 6,762,856 | 55,517,002 | 1,601 | 148,812 |
| **14F0** | F0 | 14 | 6,763,572 | 57,957,367 | 3,465 | 434,374 |
| **15F0** | F0 | 15 | 6,760,230 | 94,116,652 | 1,249 | 337,881 |
| **16F0** | F0 | 16 | 6,762,880 | 98,099,413 | 1,589 | 185,427 |
| **1F1** | F1 | 1 | 6,749,170 | 107,359,637 | 2,331 | 339,812 |
| **2F1** | F1 | 2 | 6,763,158 | 89,929,438 | 1,076 | 188,424 |
| **3F1** | F1 | 3 | 6,763,997 | 101,358,262 | 1,693 | 381,510 |
| **4F1** | F1 | 4 | 6,763,565 | 72,528,185 | 2,511 | 422,394 |
| **5F1** | F1 | 5 | 6,763,687 | 86,141,906 | 2,498 | 230,323 |
| **6F1** | F1 | 6 | 6,760,648 | 91,515,702 | 4,430 | 257,025 |
| **7F1** | F1 | 7 | 6,762,012 | 87,558,094 | 4,533 | 551,204 |
| **8F1** | F1 | 8 | 6,760,674 | 67,893,081 | 1,318 | 360,598 |
| **9F1** | F1 | 9 | 6,754,942 | 60,872,436 | 4,433 | 343,714 |
| **10F1** | F1 | 10 | 6,763,436 | 93,742,679 | 1,471 | 492,536 |
| **11F1** | F1 | 11 | 6,758,394 | 98,748,518 | 1,218 | 147,992 |
| **12F1** | F1 | 12 | 6,762,549 | 95,703,213 | 1,883 | 569,101 |

## Supplemental Table S3 cont'd

| | | | | | | |
|---|---|---|---|---|---|---|
| **13F1** | F1 | 13 | 6,762,797 | 53,968,796 | 1,634 | 252,023 |
| **14F1** | F1 | 14 | 6,763,292 | 70,708,404 | 2,179 | 272,631 |
| **15F1** | F1 | 15 | 6,761,152 | 91,424,771 | 1,298 | 239,217 |
| **16F1** | F1 | 16 | 6,763,626 | 91,778,142 | 1,535 | 306,862 |
| **1F28** | F28 | 1 | 6,747,041 | 110,265,992 | 2,084 | 384,451 |
| **2F28** | F28 | 2 | 6,763,805 | 78,515,157 | 1,349 | 201,984 |
| **3F28** | F28 | 3 | 6,764,249 | 81,750,725 | 2,511 | 307,209 |
| **4F28** | F28 | 4 | 6,763,792 | 72,750,082 | 2,515 | 464,327 |
| **5F28** | F28 | 5 | 6,764,222 | 88,617,977 | 2,488 | 339,772 |
| **6F28** | F28 | 6 | 6,762,192 | 90,549,814 | 4,752 | 570,885 |
| **7F28** | F28 | 7 | 6,764,130 | 90,120,282 | 5,569 | 356,533 |
| **8F28** | F28 | 8 | 6,762,965 | 65,060,741 | 1,079 | 563,453 |
| **9F28** | F28 | 9 | 6,734,112 | 66,329,318 | 3,148 | 163,253 |
| **10F28** | F28 | 10 | 6,762,405 | 90,316,754 | 1,437 | 246,435 |
| **11F28** | F28 | 11 | 6,752,681 | 86,885,619 | 1,304 | 196,830 |
| **12F28** | F28 | 12 | 6,758,397 | 94,661,520 | 1,836 | 371,293 |
| **13F28** | F28 | 13 | 6,761,382 | 83,573,595 | 1,079 | 348,663 |
| **14F28** | F28 | 14 | 6,763,456 | 65,101,671 | 1,895 | 323,984 |
| **15F28** | F28 | 15 | 6,761,477 | 88,632,315 | 1,130 | 466,403 |
| **16F28** | F28 | 16 | 6,762,948 | 88,755,813 | 1,535 | 132,163 |
| **1S0** | S0 | 1 | 6,758,328 | 127,951,521 | 1,953 | 130,772 |
| **2S0** | S0 | 2 | 6,764,123 | 82,944,508 | 1,365 | 359,559 |
| **3S0** | S0 | 3 | 6,764,278 | 89,974,540 | 1,735 | 196,149 |
| **4S0** | S0 | 4 | 6,764,413 | 84,031,154 | 2,052 | 307,229 |
| **5S0** | S0 | 5 | 6,764,550 | 88,782,629 | 2,055 | 377,347 |
| **6S0** | S0 | 6 | 6,761,458 | 97,078,021 | 6,156 | 339,172 |
| **7S0** | S0 | 7 | 6,764,089 | 89,195,794 | 5,526 | 342,387 |
| **8S0** | S0 | 8 | 6,763,099 | 67,389,380 | 1,527 | 281,563 |
| **9S0** | S0 | 9 | 6,759,455 | 58,761,694 | 6,742 | 498,810 |
| **10S0** | S0 | 10 | 6,762,994 | 96,063,090 | 2,056 | 531,979 |
| **11S0** | S0 | 11 | 6,757,354 | 104,894,532 | 1,524 | 323,515 |
| **12S0** | S0 | 12 | 6,760,741 | 91,735,170 | 2,552 | 666,167 |
| **13S0** | S0 | 13 | 6,763,139 | 90,366,798 | 1,240 | 265,292 |
| **14S0** | S0 | 14 | 6,763,483 | 65,994,504 | 2,451 | 290,496 |
| **15S0** | S0 | 15 | 6,763,132 | 101,206,424 | 1,001 | 370,298 |
| **16S0** | S0 | 16 | 6,763,931 | 99,210,538 | 1,581 | 343,845 |
| **1S1** | S1 | 1 | 6,758,715 | 126,658,874 | 2,185 | 99,029 |
| **2S1** | S1 | 2 | 6,764,109 | 84,433,091 | 1,348 | 436,834 |
| **3S1** | S1 | 3 | 6,764,240 | 92,764,089 | 1,643 | 232,534 |
| **4S1** | S1 | 4 | 6,764,437 | 78,793,356 | 2,003 | 306,384 |
| **5S1** | S1 | 5 | 6,764,548 | 88,992,920 | 1,941 | 464,483 |
| **6S1** | S1 | 6 | 6,761,625 | 99,661,288 | 4,882 | 266,711 |
| **7S1** | S1 | 7 | 6,764,116 | 89,505,551 | 4,607 | 201,071 |
| **8S1** | S1 | 8 | 6,763,263 | 68,459,681 | 1,481 | 326,852 |
| **9S1** | S1 | 9 | 6,759,523 | 61,776,685 | 9,132 | 423,095 |
| **10S1** | S1 | 10 | 6,763,174 | 95,518,424 | 1,962 | 302,893 |
| **11S1** | S1 | 11 | 6,757,931 | 104,591,990 | 1,574 | 255,349 |
| **12S1** | S1 | 12 | 6,762,914 | 92,535,338 | 2,346 | 461,659 |
| **13S1** | S1 | 13 | 6,762,898 | 94,660,197 | 1,302 | 401,408 |

## Supplemental Table S3 cont'd

| | | | | | | |
|---|---|---|---|---|---|---|
| **14S1** | S1 | 14 | 6,763,761 | 62,876,815 | 1,878 | 306,861 |
| **15S1** | S1 | 15 | 6,763,258 | 102,708,875 | 988 | 611,412 |
| **16S1** | S1 | 16 | 6,763,898 | 100,542,883 | 1,469 | 324,109 |
| **1S28** | S28 | 1 | 6,756,987 | 123,963,729 | 2,184 | 347,814 |
| **2S28** | S28 | 2 | 6,763,911 | 80,578,318 | 1,642 | 143,603 |
| **3S28** | S28 | 3 | 6,764,212 | 88,139,626 | 1,902 | 306,150 |
| **4S28** | S28 | 4 | 6,764,405 | 74,676,856 | 3,144 | 272,104 |
| **5S28** | S28 | 5 | 6,764,535 | 94,106,050 | 1,644 | 339,358 |
| **6S28** | S28 | 6 | 6,761,578 | 94,357,330 | 6,109 | 654,454 |
| **7S28** | S28 | 7 | 6,762,071 | 85,350,240 | 3,286 | 364,574 |
| **8S28** | S28 | 8 | 6,763,042 | 79,355,842 | 1,040 | 347,205 |
| **9S28** | S28 | 9 | 6,760,522 | 61,010,633 | 11,366 | 102,166 |
| **10S28** | S28 | 10 | 6,762,842 | 99,013,378 | 1,608 | 384,385 |
| **11S28** | S28 | 11 | 6,758,972 | 100,609,435 | 1,632 | 278,698 |
| **12S28** | S28 | 12 | 6,761,152 | 107,649,527 | 1,373 | 343,194 |
| **13S28** | S28 | 13 | 6,762,983 | 94,188,864 | 1,333 | 379,193 |
| **14S28** | S28 | 14 | 6,763,853 | 65,471,122 | 1,657 | 305,052 |
| **15S28** | S28 | 15 | 6,763,363 | 101,051,271 | 969 | 492,302 |
| **16S28** | S28 | 16 | 6,763,676 | 97,865,044 | 1,699 | 282,599 |

**Sequencing Assembly Metrics.** Sequencing assembly metrics were tabulated for each sample after normalization of number of non-human passed reads.

| Supplemental Table S4. Sequencing metrics: Wilcoxon Rank Sum analysis results | | |
|---|---|---|
| **Metric** | **Comparison** | ***p*-value** |
| **% Human reads** | Basal vs Frozen | 0.429374065 |
| **% Human reads** | Basal vs Stabilized | 0.067693846 |
| **% Human reads** | Frozen vs Stabilized | 0.005764041 |
| **% Bacterial reads** | Basal vs Frozen | 0.208298271 |
| **% Bacterial reads** | Basal vs Stabilized | 0.693106065 |
| **% Bacterial reads** | Frozen vs Stabilized | 0.239744887 |
| **% Archaea reads** | Basal vs Frozen | 0.916121258 |
| **% Archaea reads** | Basal vs Stabilized | 0.342521075 |
| **% Archaea reads** | Frozen vs Stabilized | 0.84646764 |
| **% Eukaryota reads** | Basal vs Frozen | 0.704176543 |
| **% Eukaryota reads** | Basal vs Stabilized | 0.543303716 |
| **% Eukaryota reads** | Frozen vs Stabilized | 0.764954314 |
| **% Virus reads** | Basal vs Frozen | 0.629124013 |
| **% Virus reads** | Basal vs Stabilized | 0.777214236 |
| **% Virus reads** | Frozen vs Stabilized | 0.950003308 |
| **Low quality reads** | Basal vs Frozen | 0.316201118 |
| **Low quality reads** | Basal vs Stabilized | 0.290768656 |
| **Low quality reads** | Frozen vs Stabilized | 0.034736098 |
| **Duplicated reads** | Basal vs Frozen | 0.188368101 |
| **Duplicated reads** | Basal vs Stabilized | 0.029644929 |
| **Duplicated reads** | Frozen vs Stabilized | 0.001394051 |
| **Contig length** | Basal vs Frozen | 0.32903273 |
| **Contig length** | Basal vs Stabilized | 0.271710278 |
| **Contig length** | Frozen vs Stabilized | 0.098499076 |
| **Longest contig** | Basal vs Frozen | 0.359364398 |
| **Longest contig** | Basal vs Stabilized | 0.47130121 |
| **Longest contig** | Frozen vs Stabilized | 0.341523888 |

**Statistical analysis of Sequencing Assembly Metrics.** Wilcoxon Ranked Sum test was used to generating *p*-values for pair-wise comparisons of sequencing assembly metrics across sample types, using all samples.

| Supplemental Table S5. Phyla Fold change relative to Basal | | | | | | | | | |
|---|---|---|---|---|---|---|---|---|---|
| **Phyla** | **F0 Fold Change** | **F1 Fold Change** | **F28 Fold Change** | **S0 Fold Change** | **S1 Fold Change** | **S28 Fold Change** | **F0 vs S0 *p*-value** | **F1 vs S1 *p*-value** | **F28 vs S28 *p*-value** |
| **Firmicutes** | -0.01 | -0.62 | 0.04 | 0.09 | 0.15 | 0.21 | 0.6772 | 0.03864 | 0.0155 |
| **Firmicutes** | 0.04 | 0.07 | -0.30 | -0.04 | 0.00 | -0.02 | | | |
| **Firmicutes** | -0.12 | 0.21 | -0.12 | 0.08 | 0.08 | 0.07 | | | |
| **Firmicutes** | -0.14 | -0.42 | -0.40 | 0.14 | -0.13 | 0.08 | | | |
| **Firmicutes** | -0.26 | -0.33 | -0.39 | -0.28 | -0.21 | -0.08 | | | |
| **Firmicutes** | -0.16 | -0.07 | -0.28 | -0.10 | -0.01 | -0.07 | | | |
| **Firmicutes** | 0.23 | 0.31 | 0.52 | 0.40 | 0.40 | 0.29 | | | |
| **Firmicutes** | -0.26 | 0.05 | -0.03 | 0.33 | 0.54 | 0.60 | | | |
| **Firmicutes** | -0.09 | -0.06 | 0.16 | -0.14 | 0.16 | 0.18 | | | |
| **Firmicutes** | 0.04 | 0.00 | -0.12 | 0.15 | 0.10 | 0.25 | | | |
| **Firmicutes** | -0.38 | 0.17 | -0.19 | 0.29 | 0.32 | 0.23 | | | |
| **Firmicutes** | 0.03 | 0.49 | 0.32 | 0.30 | 0.38 | 0.45 | | | |
| **Firmicutes** | 0.07 | -0.03 | 0.90 | 1.26 | 1.26 | 1.36 | | | |
| **Firmicutes** | 0.06 | 0.89 | 0.65 | 0.38 | 0.25 | -0.06 | | | |
| **Firmicutes** | 0.01 | -0.07 | 0.11 | 0.26 | 0.31 | 0.35 | | | |
| **Firmicutes** | 0.05 | -0.26 | -0.31 | 0.33 | 0.27 | 0.30 | | | |
| **Bacteroidetes** | 0.00 | 0.57 | -0.07 | -0.13 | -0.21 | -0.31 | 0.06396 | 0.4697 | 0.01825 |
| **Bacteroidetes** | -0.03 | -0.06 | 0.16 | 0.03 | 0.00 | 0.02 | | | |
| **Bacteroidetes** | 0.06 | -0.21 | 0.07 | -0.06 | -0.07 | -0.05 | | | |
| **Bacteroidetes** | 0.05 | 0.12 | 0.11 | -0.04 | 0.05 | 0.00 | | | |
| **Bacteroidetes** | 0.11 | 0.14 | 0.15 | 0.12 | 0.10 | 0.05 | | | |
| **Bacteroidetes** | 0.09 | 0.05 | 0.13 | 0.06 | 0.02 | 0.06 | | | |
| **Bacteroidetes** | -0.14 | -0.20 | -0.36 | -0.27 | -0.27 | -0.17 | | | |
| **Bacteroidetes** | 0.09 | -0.01 | 0.02 | -0.14 | -0.26 | -0.30 | | | |
| **Bacteroidetes** | 0.01 | -0.06 | -0.08 | 0.06 | -0.01 | -0.11 | | | |
| **Bacteroidetes** | -0.02 | 0.00 | 0.06 | -0.08 | -0.05 | -0.14 | | | |
| **Bacteroidetes** | 0.14 | -0.10 | 0.08 | -0.15 | -0.17 | -0.11 | | | |
| **Bacteroidetes** | 0.06 | -0.42 | -0.16 | -0.07 | -0.14 | -0.17 | | | |
| **Bacteroidetes** | -0.02 | 0.01 | -0.37 | -0.63 | -0.64 | -0.73 | | | |
| **Bacteroidetes** | -0.02 | -0.60 | -0.39 | -0.17 | -0.11 | 0.02 | | | |
| **Bacteroidetes** | 0.00 | 0.18 | 0.02 | -0.28 | -0.35 | -0.40 | | | |
| **Bacteroidetes** | -0.03 | 0.11 | 0.14 | -0.21 | -0.16 | -0.18 | | | |

**Phyla Fold Change relative to Basal.** A list of the fold changes of Bacteroidetes and Firmicutes phyla relative to Basal for Frozen (F) and Stabilized (S) samples across the time course. F0, F1, F28: Frozen Day 0, Day 1, and Day 28; S0, S1, S28: Stabilized Day 0, Day 1, and Day 28.

**Supplemental Table S6. Richness and SDI scores and statistical analysis results**

| Timepoint | Subject | Basal SDI | Basal richness | Frozen SDI | Frozen richness | Stabilized SDI | Stabilized richness |
|---|---|---|---|---|---|---|---|
| Day 0 | 1 | 4.49 | 309 | 4.53 | 315 | 4.59 | 321 |
| Day 0 | 2 | 4.09 | 280 | 4.12 | 278 | 4.10 | 282 |
| Day 0 | 3 | 4.14 | 266 | 4.14 | 260 | 4.15 | 265 |
| Day 0 | 4 | 4.11 | 239 | 4.06 | 233 | 4.15 | 237 |
| Day 0 | 5 | 3.96 | 245 | 3.84 | 232 | 3.88 | 243 |
| Day 0 | 6 | 4.18 | 258 | 4.10 | 249 | 4.19 | 251 |
| Day 0 | 7 | 3.92 | 218 | 4.03 | 237 | 4.06 | 238 |
| Day 0 | 8 | 3.41 | 284 | 3.19 | 271 | 3.43 | 280 |
| Day 0 | 9 | 3.05 | 191 | 3.00 | 189 | 3.02 | 184 |
| Day 0 | 10 | 4.13 | 264 | 4.14 | 265 | 4.21 | 274 |
| Day 0 | 11 | 4.02 | 273 | 3.85 | 261 | 4.15 | 274 |
| Day 0 | 12 | 3.89 | 243 | 3.86 | 238 | 3.93 | 261 |
| Day 0 | 13 | 3.10 | 250 | 3.19 | 257 | 3.92 | 294 |
| Day 0 | 14 | 3.62 | 229 | 3.62 | 219 | 3.75 | 239 |
| Day 0 | 15 | 4.16 | 302 | 4.15 | 302 | 4.23 | 307 |
| Day 0 | 16 | 4.19 | 284 | 4.21 | 285 | 4.30 | 296 |
| Day 1 | 1 | | | 4.44 | 306 | 4.54 | 318 |
| Day 1 | 2 | | | 4.25 | 302 | 4.15 | 286 |
| Day 1 | 3 | | | 4.35 | 290 | 4.21 | 273 |
| Day 1 | 4 | | | 3.99 | 228 | 4.04 | 231 |
| Day 1 | 5 | | | 3.96 | 243 | 3.88 | 242 |
| Day 1 | 6 | | | 4.11 | 257 | 4.21 | 257 |
| Day 1 | 7 | | | 4.08 | 256 | 4.07 | 243 |
| Day 1 | 8 | | | 3.38 | 285 | 3.47 | 271 |
| Day 1 | 9 | | | 2.98 | 194 | 3.12 | 192 |
| Day 1 | 10 | | | 4.13 | 265 | 4.20 | 270 |
| Day 1 | 11 | | | 4.13 | 281 | 4.16 | 272 |
| Day 1 | 12 | | | 4.00 | 262 | 3.95 | 260 |
| Day 1 | 13 | | | 3.14 | 258 | 4.00 | 303 |
| Day 1 | 14 | | | 3.82 | 254 | 3.71 | 237 |
| Day 1 | 15 | | | 4.12 | 300 | 4.24 | 307 |
| Day 1 | 16 | | | 4.11 | 272 | 4.30 | 295 |
| Day 28 | 1 | | | 4.47 | 311 | 4.54 | 314 |
| Day 28 | 2 | | | 3.99 | 274 | 4.08 | 278 |
| Day 28 | 3 | | | 4.01 | 257 | 4.11 | 268 |
| Day 28 | 4 | | | 3.98 | 229 | 3.93 | 220 |
| Day 28 | 5 | | | 3.86 | 232 | 3.81 | 238 |
| Day 28 | 6 | | | 4.09 | 248 | 4.11 | 245 |
| Day 28 | 7 | | | 4.01 | 242 | 4.07 | 245 |
| Day 28 | 8 | | | 3.30 | 286 | 3.54 | 277 |
| Day 28 | 9 | | | 3.12 | 205 | 3.00 | 182 |
| Day 28 | 10 | | | 4.11 | 265 | 4.19 | 272 |
| Day 28 | 11 | | | 3.88 | 261 | 4.12 | 268 |
| Day 28 | 12 | | | 3.97 | 252 | 4.26 | 297 |
| Day 28 | 13 | | | 3.78 | 290 | 3.95 | 301 |
| Day 28 | 14 | | | 3.76 | 243 | 3.68 | 224 |
| Day 28 | 15 | | | 4.09 | 295 | 4.20 | 308 |
| Day 28 | 16 | | | 4.07 | 275 | 4.29 | 296 |

| Metric | Comparison | Statistical Test | *p*-value |
|---|---|---|---|
| **Diversity** | Frozen vs Time | 1-way ANOVA | 0.551504762 |
| **Diversity** | Stabilized vs Time | 1-way ANOVA | 0.968167644 |
| **Richness** | Frozen vs Time | 1-way ANOVA | 0.103835023 |
| **Richness** | Stabilized vs Time | 1-way ANOVA | 0.96475062 |
| **Richness** | Frozen vs Stabilized | Rank Sum | 0.106944848 |
| **Diversity** | Frozen vs Stabilized | Rank Sum | 0.001711375 |

**Statistical analysis of Diversity.** SDI and richness scores are listed for each sample at each timepoint and treatment. 1-way ANOVA or Wilcoxon Rank Sum tests, as listed, were used to generate p-values comparing richness and diversity scores across sample types.
